# The Use of Diagnostic Tumor Markers in Detecting Tobacco‐ and Betel Quid‐Induced Oral Squamous Cell Carcinoma: A Scoping Review of Empirical Evidence

**DOI:** 10.1002/hsr2.70650

**Published:** 2025-04-18

**Authors:** Yovanthi Anurangi Jayasinghe, Kalpani Senevirathna, Afeez Abolarinwa Salami, Kehinde Kazeem Kanmodi, Ruwan Duminda Jayasinghe

**Affiliations:** ^1^ Department of Oral Medicine and Periodontology, Faculty of Dental Sciences University of Peradeniya Peradeniya Sri Lanka; ^2^ Centre for Research in Oral Cancer, Faculty of Dental Sciences University of Peradeniya Peradeniya Sri Lanka; ^3^ Department of Biochemistry, Faculty of Medicine Uva Wellassa University of Sri Lanka Badulla Sri Lanka; ^4^ Faculty of Dentistry, University of Puthisastra Phnom Penh Cambodia; ^5^ Department of Oral and Maxillofacial Surgery University College Hospital Ibadan Nigeria; ^6^ Department of Public Health Dentistry Manipal Academy of Higher Education Manipal India; ^7^ School of Dentistry, University of Rwanda Kigali Rwanda; ^8^ Campaign for Head and Neck Cancer Education (CHANCE) Programme Cephas Health Research Initiative Inc Ibadan Nigeria; ^9^ School of Health and Life Sciences, Teesside University Middlesbrough UK; ^10^ School of Public Health, University of Port Harcourt Port Harcourt Nigeria

**Keywords:** betel quid, biomarkers, oral squamous cell carcinoma, review, tobacco

## Abstract

**Background and Aims:**

Tobacco and betel quid are two major causes of oral squamous cell carcinoma (OSCC). Tumor biomarkers offer potential headway for improved diagnosis of OSCC caused by tobacco and betel quid. Currently, several empirical investigations have explored the use of diverse types of tumor biomarkers in the diagnosis of tobacco‐ and betel quid‐induced OSCC; however, no known study has mapped the evidence reported in those studies. This scoping review aims to map existing empirical evidence on the biomarkers of OSCC caused by these carcinogenic substances.

**Methods:**

This scoping review adhered to the PRISMA‐ScR checklist. Seven databases (PubMed, SCOPUS, AMED, APA PsycArticles, APA PsycINFO, CINAHL Ultimate, Dentistry, and Oral Sciences Source) were searched to retrieve literature on diagnostic tumor markers used in detecting tobacco‐ and betel quid‐induced OSCC. After deduplication and screening of research articles based on the eligibility criteria. Only 36 peer‐reviewed articles met the inclusion criteria of the scoping review. Data from the selected articles were charted, collated, summarized, and presented.

**Results:**

All the included articles were on studies published within the past decade. Most of them (26 articles) reported case‐control studies. More than half (20 articles) were on studies conducted in India. A total of 6707 participants were investigated in the included articles, and different oral anatomical sites were involved. The biomarkers investigated were of diverse types, ranging from genomic biomarkers to transcriptomic markers. Specifically, these markers include micronuclei, miRNAs, protein markers, gene alterations, and salivary markers. These biomarkers were used in the early detection, risk assessment, and prognosis evaluation of tobacco‐/betel quid‐induced OSCC.

**Conclusion:**

This scoping review provides insights into the current global research landscape on the use of biomarkers in the diagnosis of tobacco‐ and betel quid‐induced OSCC; it also provides potential avenues for improving early detection and management of this prevalent oral malignancy.

## 1 Introduction

Oral squamous cell carcinoma (OSCC) belongs to the broader spectrum of head and neck cancers, and it is the most prevalent type of oral cancer, primarily impacting the cell lining of the oral cavity [1, 2]. According to the Global Cancer Statistics reports in 2020, OSCC is ranked as the 18th most prevalent malignancy worldwide, reporting an annual incidence of 377,713 cases and 177,757 incidences of mortality with a significant impact on public health [3]. Smoking and smokeless tobacco, consumption of betel quid along with betel leaf together with areca nut and slaked lime [4], excessive consumption of alcohol [5], and human papillomavirus (HPV) infection [6] are the primary etiological factors contributing to the progression of OSCC. However, based on recent published literature, the majority of tumors that occur in the oropharynx with previous HPV infections are categorized as HPV‐positive HNSCC, whereas tumors that occur in the oral cavity and larynx are classified as HPV‐negative HNSCC as most are associated with smoking [7, 8]. Prolonged exposure to these risk factors escalates tumorigenesis due to its carcinogenic substances, leading to genetic alterations and mutations in oral tissues [9]. Early detection of OSCC is essential to improve patient outcomes and reduce the burden of this disease. Conventional diagnostic approaches, such as clinical examination, histopathology, and imaging techniques, have been the gold standard for many years. However, these methods often lack the sensitivity and specificity required to identify OSCC at its nascent stages when it is most treatable.

In response to the development of cancer, it can produce abnormal cellular substances, which are associated with malignant neoplastic cells known as “tumor markers” [10]. These tumor markers are either produced by the body itself or by the cancer cells, which can be detected in different bodily fluids such as blood, urine, serum, saliva, and on the surface of cancer cells through biochemical methods or immunohistochemistry [10]. Tumor markers possess extensive applications in diagnosing, monitoring treatments, and predicting cancer progression. Based on its presence, tumor markers are divided into Tumor‐specific and Tumor‐associated markers [11]. Alterations due to tumor progression do not accurately reflect on clinicopathological images; thus, tumor markers are valuable in aiding cancer diagnosis and management due to their sensitivity and specificity. Modest sensitivity and specificity of tumor markers were proved by multiple studies [12, 13, 14] about OSCC.

Exposure to tobacco use and betel quid chewing are prone to induce specific molecular changes and alterations in gene expression patterns within the oral cavity. These changes allow upregulation and downregulation of certain proteins and biomolecules [15]. Although different studies, including reviews, have been conducted on the roles of tumor markers in diagnosing and predicting cancer outcomes induced by tobacco and betel quid use [16, 17]. Yet, there has not been a recent scoping review to systematically outline the existing evidence on the roles of diagnostic tumor markers in detecting tobacco‐ or betel quid‐induced OSCC. This scoping review was conducted to identify and map existing scientific knowledge gaps with empirical evidence on tumor markers on tobacco‐ and betel quid‐induced OSCC.

## 2 Methods

### 2.1 Research Design

We conducted this scoping review based on the methodological framework designed by Arksey and O'Malley, (2005) [18], and the Preferred Reporting Items for Systematic Reviews and Meta‐Analyses extension for Scoping Reviews (PRISMA‐ScR) checklist modified by Tricco *et al*. (2018) [19] was adopted for the reporting process.

### 2.2 Identification of Research Question

This research question was addressed in this scoping review: What empirical evidence exists on the use of diagnostic markers in detecting tobacco‐ and betel quid‐induced OSCC?

### 2.3 Identification of Relevant Literature

In this scoping review, multiple literature sources of research databases and reference lists of the included literature were used to identify relevant literature. A comprehensive and systematic search of seven research databases, including PubMed, SCOPUS, CINAHL Ultimate, Allied and Complementary Medicine Database (AMED), APA PsycArticles, APA PsycINFO, and Dentistry and Oral Sciences Source, was conducted on July 27, 2023. The search aimed to gather literature pertinent to the scoping review question, employing a combination of specific search terms together with Boolean operators (“AND” and “OR”) and truncations (“*”). The search strings used for the search are shown in Supporting Information S1: Tables S1–S3.

Furthermore, to ensure that the literature search strategy is comprehensive, we manually searched the reference lists of the included literature to identify any other literature that was not identified from the research database search. Notably, this was done after the eligible literature have been selected from the pool of literature retrieved from the research database search.

### 2.4 Selection of Eligible Literature

After the literature search, all literature retrieved from the searched databases was imported into the Rayyan web application for deduplication [20], where all the duplicate records were deleted. The deduplicated literature was then screened by four reviewers (Y.A.J., K.S., A.A.S., K.K.K.) in a two‐stage process for inclusion/exclusion into this scoping review. In stage 1, screening of titles and abstracts was conducted to exclude literature which were not within the inclusion criteria of this scoping review. In contrast, a full‐text screening of the initially included literature was done in the second stage. All discrepancies and conflicts were resolved through comprehensive discussion among the four reviewers. Only those literature meeting the specified criteria below were taken into account for inclusion or exclusion in this scoping review:

#### 2.4.1 Inclusion Criteria

- Literature published in peer‐reviewed journals.
- Literature reporting experimental findings on the use of diagnostic tumor markers in detecting tobacco‐ or betel quid‐induced OSCC.
- Literature published in the English language.
- Literature with available full text.

#### 2.4.2 Exclusion Criteria

- Literature published in non‐peer‐reviewed journals such as books, book chapters, webpages, etc.
- Peer‐reviewed journal literature that are not original research articles (e.g., review articles, editorials, letters, commentaries, etc).
- Literature published in languages other than English.
- Literature that does not report experimental findings on the use of diagnostic tumor markers in detecting tobacco‐ or betel quid‐induced OSCC.
- Literature without available full text.

### 2.5 Data Charting, Collation, and Summarization

The included literature was subjected to data extraction using a customized data extraction template. This included information on authors' names, publication year, study design, country, sample size, sample characteristics, cancer sites, risk habits (tobacco/betel quid or other), biomarker, key findings, and conclusions. The extracted data were collated, summarized, and presented through written content and tables.

### 2.6 Risk of Bias Assessment

Given the diversity of study designs among the included articles, the Mixed Method Appraisal Tool was utilized to assess the risk of bias for this scoping review. Consequently, all the included articles were critically appraised by two independent reviewers. The appraisal tool comprised of seven points, which include two generic screening questions and five signaling questions for each study design. Each question consists of three responses each, which are “Yes” receiving a score of one (1), “I can't tell” receiving a score of 0.5, and “No” receiving a score of 0. Based on the responses, the total score of each included article ranged between 0 and 7. Based on the total score, the quality of each study was appraised as “above average” if the total score was ≥ 4, “average” if the score was 3.5, or “below average” if the total score was ≤ 3 (Supporting Information S1: Tables S5–S8).

### 2.7 Ethical Considerations

Being a scoping review, where no primary data was obtained from human or animal subjects, obtaining ethical clearance and consent for this study was not applicable.

## 3 Results

**Figure 1:**
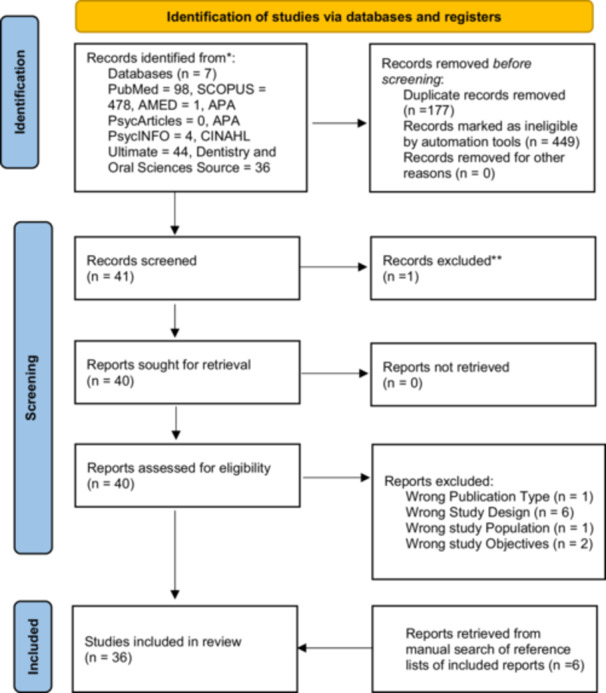
PRISMA flowchart diagram.

Six hundred and sixty‐one literature sources were retrieved from multiple databases (PubMed = 98, SCOPUS = 478, The Allied and Complementary Medicine Database (AMED) = 1, APA PsycArticles = 0, APA PsycINFO = 4, CINAHL Ultimate = 44, Dentistry and Oral Sciences Source = 36. Among these, 171 duplicate records were detected and subsequently removed. From the remaining 490 deduplicated literature, 449 were excluded following title and abstract screening. Forty‐one pieces of literature were subjected to full‐text evaluation, after which only 21 were within inclusion criteria of this scoping review (Supporting Information S1: Table S4, Figure 1). After conducting a thorough manual search of the literature provided, 1228 literature were reviewed for eligibility. Following this, 7 duplicates were identified and removed. Subsequently, 1136 literature were excluded based on the screening of their titles and abstracts. Eighty literature were subjected to full‐text evaluation and only 30 met the eligibility criteria of this scoping review (Figure 1). After the manual search of the reference lists of these articles, 6 additional relevant articles were obtained. Thereby, a total of 36 research articles (papers) were finally included in this scoping review.

### 3.1 Publication Type and Publication Trend by Year

All the included literature were original research articles published within a decade till 2023. Among them, eight were published in 2021 [21, 22, 23, 24, 25, 26, 27, 28], five each in 2020 [29, 30, 31, 32, 33] and 2023 [34, 35, 36, 37, 38], four in 2019 [39, 40, 41, 42], three each in 2016 [43, 44, 45], 2017 [46, 47, 48] and 2022 [49, 50, 51], two each in 2013 [52, 53], 2014 [54, 55] and one 2018 [56] (Table 1).

**Table 1:** Summary of the study characteristics.

No.	Author (year)	Study design	Country	Sample size	Age range	Tobacco/betel quid or other risk habits	Sample characteristics	
							Study cohorts	Selection criteria
1.	Patel et al. (2021) [21]	Cohort study (Prospective)	India	400	15 to 75	Tobacco and related habits	Group 1: Individuals exhibiting tobacco and related habits, and habit‐related oral lesions, yet without a diagnosis of OSCC. Group 2: Individuals with tobacco and related habits but without any associated oral lesions due to these habits. Group 3 (Control group): Individuals with a history of tobacco use andrelated habits who have been diagnosed with oral cancer. Group 4 (Control group): Healthy individuals without any habits or habit‐related oral lesions who are in good health.	**Inclusion criteria** Individuals who have maintained tobacco use and related habits for a minimum of the past 5 years. Individuals with oral lesions associated with tobacco use or diagnosed with oral cancer due to tobacco‐related causes. **Exclusion criteria:** Individuals with oral lesions that are distinct from those caused by tobacco use. Individuals who have undergone treatment for oral lesions specifically linked to tobacco use in the past. Individuals with any with any underlying systemic illness or condition. Individuals who have undergone a radiographic procedure within the past month. Individuals who are exposed to known DNA‐damaging agents or ionizing radiation.
2.	Sajid et al. (2023) [34]	Cohort study (Prospective)	India	44	Information not stated	SLT consumers Alcohol	Group 1: Individuals who do not consume smokeless tobacco (SLT) (control group) Group 2: Individuals who are SLT consumers without oral premalignant lesions (OLP) Group 3: Individuals who are SLT consumers presented with OPL Group 4: Individuals who are SLT alcohol consumers presented with OPL	**Inclusion criteria:**Individuals who were not on any medication for three months before sample collection were included in the study
3.	Chen et al. (2021) [22]	Case‐control study	China	197	20 ‐ 83	Smoking Alcohol	Group 1: OPMD group (leukoplakia, erythroplakia, lichen planus) Group 2: OSCC patients Group 3: Healthy volunteers	Cancer‐free and OPMD‐free were included as the control group.
4.	Singh et al. (2018) [56]	Case‐control study	India	90	Information not stated	Tobacco Chewers Smoking Pan Masala Alcohol	Group 1: Individuals with Oral sub‐mucous fibrosis (OSMF) Group 2: Individuals with OSCCGroup 3: Healthy volunteers (Control group)	**Inclusion Criteria** All newly diagnosed cases of OSCC, OSMF, and healthy controls who agreed to partake in the study. **Exclusion Criteria** Cases who are present with or had any malignancies in the past. Patients who have known Immunodeficiency disorders such as AIDS. Individuals in the advanced stage of the disease who were not eligible for surgery.
5.	Ren et al. (2014) [54]	Case‐control study	China	90	25–92	Smoking	Individuals with histological diagnosis of OSCC Volunteers who were cancer‐free donors matched with age and sex.	The control group consisted of individuals who were enrolled at the same institute and had no prior history of OSCC for over two years.
6.	Rochefort et al. (2022) [49]	Cohort study (Prospective)	France	87	28–87	Smoking, Alcohol	Group 1: OSCC patients who are nonsmokers or non‐drinkers and OSCC patients who are smokers or drinkers Group 2: Healthy volunteers who are nonsmokers or non‐drinkers and Healthy volunteers who are smokers or drinkers	OSCC patients who were HPV negative were recruited. All participants were recruited between 2013 and 2017.
7.	Amer et al. (2019) [39]	Cohort study (Prospective)	Egypt	64	Information not stated	Shisha smokers Cigarette smokers	Group 1: Nonsmokers Group 2: Shisha smokers Group 3: Cigarette smokers Group 4: Simultaneous cigarette and shisha smokers.	**Inclusion criteria:** Participants who were not on medication. **Exclusion criteria:** Subjects who had systemic illnessSubjects exposed to carcinogens. Selection criteria of Shisha smokers: Regular shisha smokers (at least three times a week) for a duration of at least 5 years. Selection criteria of Cigarette smokers: Regular smokers (at least ten cigarettes per day) for a duration of at least 5 years. Selection criteria of Simultaneous smokers: Must meet both smoking criteria
8.	Huang et al. (2014) [55]	Case‐control study	Taiwan	71	Information not stated	Alcohol consumption Cigarette smoking Betel nut chewing	Patients diagnosed with OSCC. Malignant tumors were identified on the tongue, gums, mouth floor and other unspecified areas of the mouth. Participants with non‐neoplastic controls of similar age and gender (within ±3 years) recruited from outpatient clinics.	Individuals who abstained from alcohol within the last year or had consumed less than 12 drinks throughout their lifetime were classified as non‐drinkers.
9.	Aghiorghiesei et al. (2022) [50]	Case‐control study	Romania	33	Information not stated	Smoking	Group 1: Oral tissue samples of OSCC Group 2: Adjacent normal tissues	Information not stated
10.	Bhuvaneswari et al. (2022) [51]	Cohort study (Prospective)	India	81	20–70	Smokers	Group 1 (Control Group): Healthy individuals with normal oral mucosa based on clinical assessment Group 2: Smokers with a history of at least 3 years with normal oral mucosa based on clinical assessment Group 3: Patients diagnosed clinically with oral lesion. Group 4: Patients histopathologically diagnosed with OSCC	**Exclusion criteria:** Participants with systemic illnesses Participants with prior history of cancer, or currently undergoing chemotherapy or antioxidant therapy,
11.	Rezazadeh et al. (2017) [46]	Cohort study (Prospective)	Iran	48	Information not stated	Smoking	Patients with OSCC Healthy individuals who are non‐smokers	Individuals with any systemic illness, history of radiotherapy, chemotherapy, or other forms of cancer were not included.
12.	Vishwakarma et al. (2020) [29]	Case‐control study	India	32	23–72	Smoking Alcohol	**Selection criteria:** Group 1: Tumor group (OSCC) Group 2: Normal group	**Exclusion criteria:** Participants who had undergone radiotherapy and/or chemotherapy
13.	Arunkumar et al. (2017) [47]	Cohort study (Prospective)	India	68	Information not stated	Smokers Tobacco chewers Alcohol drinkers	Group 1: Individuals who are exclusive smokers. Group 2: Individuals who are exclusive tobacco chewers. Group 3: Individuals who are alcohol drinkers. Group 4: Individuals with mixed habits such as smoking and alcohol drinking, smoking and tobacco chewing	Information not stated
14.	Su et al. (2019) [40]	Case‐control study	Taiwan	2362	Information not stated	Betel quid chewing Cigarette smoking Alcohol drinking	Control group: healthy male individuals with no self‐reported history of any cancer. Case group: male patients with oral cancer	Information not stated
15.	Cheng et al. (2016) [43]	Cross‐sectional analytical study	Taiwan	267	Information not stated	Areca‐quid chewing Cigarette smokingAlcohol drinking	Group 1: Individuals seeking a visual oral examination (VOE) Group 2: Patients referred for additional evaluation because of abnormal VOE findings.	**Inclusion criteria:** Individuals without any disease, oral precancer, or oral cancer, aged above 20 years, and who provided signed informed consent forms (ICF). **Exclusion criteria:** pregnant, diagnosis of oral lichen planus, or a history of head and neck cancer which was previously treated
16.	Tandon et al. (2017) [48]	Case‐control study	India	200	Information not stated	Tobacco, Areca nut, Alcohol, Smoking	Case group: Patients who were clinically diagnosed and histopathologically confirmed OSCC Control group: Healthy individuals	**Inclusion criteria** Patients more than 18 years of age **Exclusion criteria** Individuals with systemic diseases Patients suffering from infectious diseases or any other cancers. Patients with known systemic nutritional deficiencies Pregnant or lactating females
17.	Mondal et al. (2013) [53]	Cohort study (Prospective)	India	260	Information not stated	Tobacco‐betel quid chewing Smoking Alcohol intake	Group 1: Patients with OSCC Group 2: Participants without OSCC, but who chew tobacco‐betel quid habitually and have no family history of cancer	Information not stated
18.	Sun et al. (2023) [35]	Cohort study (Retrospective)	China	Information not stated	Information not stated	Betel quid (Arecoline)	This study was confined to analyzing gene expression profiles, specifically focusing on *Homo sapiens* as the subject species.	Information not stated
19.	D'Cruz et al. (2021) [23]	Cohort study (Prospective)	India	15	Information not stated	Tobacco – smoking Smokeless tobacco – Betel quid chewing	Information not stated	**Inclusion criteria** Participants above 18 years of age Participants clinically diagnosed and histologically confirmed with OSCC **Exclusion criteria** Patients with a prior history of cancer and individuals who have received treatments for cancer Participants with a history of previous exposure to carcinogens, radiation or heavy metals.
20.	Sawant et al. (2023) [36]	Cohort study (Prospective)	India	120	Information not stated	Tobacco chewers	Group 1: Healthy individuals Group 2: Patients suffering from OSCC Group 3: Long‐term tobacco chewers	**Inclusion criteria** Participants who were found to have no diagnosed oral cavity disorders during previous clinical assessments were classified as healthy controls. Participants who had been using tobacco for a minimum of 5 years were classified as long‐term tobacco users. **Exclusion criteria** Individuals younger than 18 years of age Individuals who were medically compromised or unable to provide consent Participants who were fully edentulous Individuals who had undergone previous oncological treatment.
21.	Goel et al. (2021) [24]	Case‐control study	India	70	Information not stated	Smokers and tobacco chewers	Participants diagnosed with OSCC Individuals not suffering from any cancer were considered as controls	Information not stated
22.	Lepcha et al. (2021) [25]	Cohort study (Prospective)	India	50	Information not stated	Betel nut chewing	Case group— Individuals who chew raw betel nuts (non‐alcohol and tobacco) Control group—Individuals who are not exposed to risk habits; raw betel nuts, alcohol, or tobacco	Individuals of both genders aged 15 and above were included in the study.
23.	Liyanage et al. (2019) [41]	Cross‐sectional analytical study	Sri Lanka	148	Information not stated	Cigarette smoking, Betel quid chewing	Group 1: Patients who are newly diagnosed with cancer originating in the oral cavity. Group 2: Patients with oropharyngeal tumors or loco‐regional metastasis originating from the oral or oropharyngeal region.	Healthy controls consisted of individuals matched for age and gender, who had no prior history of malignancies.
24.	Ueda et al. (2021) [26]	Case‐control study	Japan	121	27 to 91	Cigarette Alcohol	Group 1: OSCC patients Group 2: OPMD patients Group 3: healthy volunteers	Information not stated
25.	Triani et al. (2021) [27]	Cohort study (Prospective)	Indonesia	34	Information not stated	Betel nut chewers	Case group: Individuals with a malignant or premalignant lesions who are betel nut chewers Control group: Healthy volunteers who are non‐betel nut chewers	Participants with informed consent were included in this study
26.	Smriti et al. (2020) [30]	Cohort study (Prospective)	India	88	26–95	Tobacco	Patients with OSCC Patients with OPMDs (Erythroplakia, Leukoplakia, OSF) Individuals with tobacco habits Healthy controls	**Inclusion criteria:** OSCC and OPMD cases should be histopathologically confirmed Individuals who were 18 years of age and above Individuals with tobacco habits more than 1 year without any clinical signs of OSCC or OPMD. Healthy individuals with no tobacco habit and other underlying health conditions were included in the study.**Exclusion criteria:** Individuals with autoimmune diseases, previous history of cancer, cancer treatments, autoimmune disorders, hepatitis or human immunodeficiency virus infections, pregnancy or lactation
27.	Patil et al. (2021) [28]	Case‐control study study	India, Saudi Arabia, UAE	100	18–60	Gutkha, Betel nut, Smoking, Alcohol	Group 1: Healthy controls Group 2: Clinically diagnosed and histopathologically confirmed oral leukoplakia Group 3: Clinically diagnosed and histopathologically confirmed OSF Group 4: Clinically diagnosed and histopathologically confirmed OSCC	**Selection criteria:** Individuals with current or previous systemic diseases (diabetes, tuberculosis, hypertension, and liver disease) Individuals who underwent cancer treatments such as radiotherapy or chemotherapy, Patients who had any form of malignancies in the past Individuals over 60 years old due to potential immunocompromised states
28.	Ukey et al. (2023) [37]	Case‐control study	India	93	20–75	Smoking Alcohol Tobacco chewing	Group 1: Histopathologically confirmed OSCC patients Group 2: Histopathologically confirmed OSMF patients Group 3: Healthy persons (Control)	**Exclusion criteria:** OSCC and OSMF patients with HPV infection OSMF patients who have a mouth opening exceeding 25 mm
29.	Azeem et al. (2020) [31]	Cohort study (Prospective)	India	96	31–60	Tobacco chewing	Group 1: Clinically diagnosed and histopathologically confirmed OSCC with tobacco chewing habit Group 2: Tobacco chewers without any malignant or premalignant oral lesions Group 3 – Healthy controls who were not exposed to tobacco habits as control group	**Exclusion criteria:** Patients with recurring or chronic inflammatory oral cavity lesions, (pemphigus/Behcet's syndrome), patients who have had radiation, oncosurgery, or neoadjuvant chemotherapy, patients with immunodeficiency, patients with mixed habits, such as alcohol abusers and tobacco smokers, were excluded from the research.
30.	Juan et al. (2023) [38]	Cohort study (Prospective)	Taiwan	171	Information not stated	Betel quid chewing Cigarette smoking Alcohol drinking	Group 1: Histopathologically confirmed benign mucosal lesions Group 2: individuals with a normal oral mucosa	**Exclusion criteria:** Patients with a history of head and neck cancer; patients receiving chemotherapy, patients having oral lichen planus and pregnant women OSCC patients who received their initial clinical examination, histological evaluation and surgical treatments within 2012 to 2014
31.	Chaudhari et al. (2016) [44]	Case‐control study	India	90	31–70	Tobacco Smokeless tobacco	Group 1: Patients with OSCC Group 2: Patients with premalignant lesions (OLK, Erythroplakia, smokers’ palate, smokeless tobacco keratosis) Group 3: Healthy control with good oral hygiene and no systematic disorders.	**Exclusion criteria:** Pregnant females Patients suffering from any systemic disease
32.	Bose et al. (2013) [52]	Cohort study (Prospective)	India	160	23–50	Tobacco Areca quid	Group 1: Healthy control without habits (Tobacco and areca quid) Group 2: Healthy control with habits (Tobacco and areca quid) without any systemic disease Group 3: Newly diagnosed oral leukoplakia (OLK) patients of both genders Group 4: Newly diagnosed oral submucous fibrosis (OSF) patients of both genders Group 5: Oral cancer patients who didn't undergo any treatments nor antioxidant therapy.	**Inclusion criteria:** Participants without any systemic illness OSCC patients, who did not receive any previous treatments nor antioxidant therapy
33.	Goyal (2020) [32]	Cohort study (Prospective)	India	500	Information not stated	Tobacco	Group 1: Healthy individuals who nontobacco users Group 2: Individuals who habitually consumed tobacco or smoked but did not exhibit any oral lesions. Group 3: Patients with benign oral lesions (Tobacco pouch keratosis, Stomatitis Nicotina, etc.) Group 4: Patients with a history of tobacco use and evident precancerous lesions (leukoplakia, erythroplakia, Lichen planus, etc.) Group 5: Patients with frank Oral Cancer	**Exclusion Criteria** Patients with systemic illness (e.g. diabetes, cardiovascular disease, muscular dystrophy, bone diseases, kidney diseases, pancreatic diseases, blood dyscrasis, liver or heapatobilliary diseases, etc.) Individuals with a medical history for drugs like anesthetics, narcotics, aspirin Postmenopausal women Patients with additional mucosal lesions and underlying local or systemic conditions that elevated levels of LDH and ALP
34.	Oh et al. (2020) [33]	Case control study	Korea	67	25–97	Smoking Alcohol	Group 1: Individuals who were not present with OSCC Group 2: OSCC patients	The majority of participants in the control group were individuals who visited the hospital for procedures such as third molar extraction or prosthetic treatment. None of the individuals in the control group exhibited precancerous lesions, such as leukoplakia.
35.	Dineshkumar et al. (2016) [45]	Case control study	India	300	21–90	Tobacco chewing Smoking Alcohol	Healthy control group PMD group OSCC group	***Healthy control group*** **Inclusion criteria:** Individuals within ages 21–65**Exclusion criteria:** Individuals currently using prescribed or non‐prescribed medication Individuals with chronic or acute illnesses Individuals with oral lesions, acute or sub‐acute inflammation or infection Individuals with pathological dry mouth syndrome Individuals encountered difficulty in obtaining adequate saliva samples consistently Pregnant and lactating women ***PMD group*** **Inclusion criteria** Individuals within ages 21–90 Patients clinically diagnosed and histopathologically confirmed with OPMDs Individuals who had not received or were not currently receiving any treatment for oral lesions **Exclusion criteria** Individuals with systemic illnesses Individuals with oral inflammatory conditions (gingivitis, periodontitis, and oral ulcers) ***OSCC group*** **Inclusion criteria:** Individuals within ages 21–90 Clinically confirmed and histopathologically diagnosed OSCC patients Individuals who were not undergoing or had not undergone any cancer treatments **Exclusion criteria:** Non‐confirmed OSCC by tissue biopsy Other systemic illness except OSCC
36	Deepthi et al. (2019) [42]	Case control study	India	90	20–74	Smokers Tobacco chewers	Group 1: Clinically and histopathologically diagnosed oral leukoplakia patients Group 2: Clinically and histopathologically diagnosed OSCC patients Group 3: age and sex matched healthy controls	**Exclusion criteria** Individuals with pre‐existing medical conditions that could elevate cytokine levels in saliva Individuals who have previously undergone local therapeutic treatments for other oral conditions. Individuals on medications (antihistamines, antihypertensives, anticholinergics, antidepressants, or bronchodilators) Pathological dry mouth syndrome Difficulty in obtaining adequate saliva samples consistently

### 3.2 Study Designs of Included Articles

Considering the study designs of the included articles, majority (94.4%) of the included articles either adopted a case‐control study design (*n *= 17/36) [22, 24, 26, 28, 29, 33, 37, 38, 40, 42, 44, 45, 48, 50, 54, 55, 56] or a cohort study design (*n *= 17/36) [21, 23, 25, 27, 30, 31, 32, 34, 35, 36, 39, 46, 47, 49, 51, 52, 53]. Only 5.6% of articles adopted a cross‐sectional analytical study design [41, 43]. Among those articles which adopted a cohort study design (*n* = 17/36), 94.1% (*n* = 16/17) of them were prospective studies [21, 23, 25, 27, 30, 31, 32, 34, 36, 39, 46, 47, 49, 51, 52, 53], whereas only 5.9% (*n* = 1/17) were retrospective studies [35] (Table 1).

### 3.3 Risk of Bias Assessment Outcomes

All the included articles (*n* = 36) were considered as quantitative non‐randomized studies, based on the Mixed Methods Appraisal Tool. The quality of all the articles appraised were above average, with their quality appraisal scores ranging between 4 and 7. The highest score of 7 was obtained among 11.1% (*n* = 4/36) of the articles [21, 32, 38, 40], whereas the lowest score of 4 was obtained by only one article (2.8%) [25]. Fourteen articles (38.9%) obtained a score of 6.5 each [22, 26, 27, 28, 29, 35, 36, 39, 43, 44, 45, 46, 47, 51], nine articles (25%) obtained a score of 5.5 each [30, 31, 37, 42, 49, 54, 55, 56, 57], five articles (13.9%) obtained a score of 5 each [23, 33, 34, 50, 52], and 3 articles (8.3%) obtained a score of 6 each [41, 48, 53] (Supporting Information S1: Tables S5–S8).

### 3.4 Study Populations and Geographical Locations

A total of 6707 participants (patients and healthy controls) were studied in these included articles, with an article having the smallest sample size of 15 persons [23] and another having the highest sample size of 2362 persons [40]. Majority of the selected articles were conducted in Asia (91.4%) [21, 22, 23, 24, 25, 26, 27, 29, 30, 31, 32, 33, 34, 35, 36, 37, 38, 40, 41, 42, 43, 44, 45, 46, 47, 48, 51, 52, 53, 54, 55, 56] and only few (3) articles were on studies conducted outside Asia (8.6%) [39, 49, 50]. Regarding the country of origin, 20 articles were on studies conducted in India (57.1%) [21, 23, 28, 29, 31, 34, 36, 37, 44, 47, 51, 52, 53], 4 were studies from Taiwan (11.4%) [38, 40, 43, 55, 56], 3 from China (8.6%) [35, 54] and one article each was from Sri Lanka [41], Egypt [39] Japan [26], France [49], Indonesia [27], Iran [46], Korea [33] and Romania [50], (22.9%) (Table 1).

### 3.5 Characteristics of Study Cohorts

From the included articles, study cohorts were classified according to histopathological features, clinical features, and risk habits. The age ranges of the participants in most of the included articles were between 20 and 75 years of age [21, 22, 26, 28, 29, 30, 31, 33, 37, 42, 44, 45, 49, 51, 52, 54], whereas the minimum and maximum ages recruited were 15 years [21] and 97 years [33]. The main risk factors assessed were smoked tobacco (83.3%) [21, 22, 23, 24, 26, 28, 29, 30, 32, 33, 37, 38, 39, 40, 41, 42, 43, 44, 45, 46, 47, 48, 49, 50, 51, 52, 53, 54, 55, 56] and smokeless tobacco (63.9%) [23, 24, 25, 27, 28, 31, 34, 35, 36, 37, 38, 40, 41, 42, 43, 44, 45, 47, 48, 52, 53, 55, 56]. Apart from that, alcohol consumption (47.2%) was also assessed in some included articles [22, 26, 28, 29, 33, 34, 37, 38, 40, 43, 45, 47, 48, 49, 53, 55, 56]. Among the tobacco users, majority were on smokers/cigarette smokers [22, 23, 24, 26, 28, 29, 33, 37, 38, 39, 40, 41, 42, 43, 45, 46, 47, 48, 49, 50, 51, 53, 54, 55, 56] and only one study was on shisha smokers [39]. Studies on smokeless tobacco were mostly on betel quid chewing (56.5%) [23, 25, 27, 28, 35, 38, 40, 41, 43, 48, 52, 53, 55] and one each on Gutka [28] and Pan masala [56] (Table 1).

### 3.6 The Tumor Markers Reported

In several of the included articles, OSCC were mainly developed in the buccal mucosa (88.2%) [21, 22, 26, 27, 29, 30, 38, 41, 42, 43, 45, 47, 48, 49, 55] and tongue (76.5%) [22, 26, 29, 30, 38, 41, 42, 45, 47, 48, 49, 50, 55]. Additionally, 47.1% were on lip [29, 41, 47], 41.2% on floor of the mouth, 29.4% of each were on hard palate [22, 29, 30, 41, 49] and gingiva [22, 26, 38, 48, 49], 23.5% on alveolar ridge [22, 47, 49, 55], 17.6% on palate [42, 45, 50], 11.8% of each on oral mucosa [39, 43], oropharynx [41, 42], alveolar mucous [42, 45], alveolus [30, 50] and retromolar trigone [30, 42] and 5.9% of each were reported on pharyngeal inlet [55], retromolar [41], gingivobuccal sulcus [29], gum [50], tonsil [50] and vestibule [45]. In most of the studies the tumor markers were assessed using saliva samples (38.9%) [23, 25, 26, 27, 30, 31, 32, 33, 39, 41, 42, 44, 45, 51], blood (30.6%) [22, 24, 32, 37, 40, 45, 48, 49, 52, 54, 56] and tissue specimens (30.6%) [23, 27, 28, 29, 35, 37, 38, 47, 49, 50, 53].

The results on tumor markers of the included articles were categorized into five groups and of all included articles, 9 articles were on genomic markers [21, 23, 24, 38, 40, 41, 43, 53, 55], 8 articles were on transcriptomic markers [29, 33, 37, 47, 48, 50, 54, 56], 11 articles were on proteomic markers [22, 25, 26, 27, 28, 35, 39, 42, 45, 46, 49], 6 articles were on metabolomic markers [30, 31, 32, 44, 51, 52] and only 2 articles were on the microbiome [34, 36]. Among the genomic markers, 3 articles were on polymorphisms [23, 40, 53], 5 articles were on DNA hypermethylation [24, 38, 41, 43, 55] and one article was on excessive formation of micronuclei [21]. Long noncoding RNA (*n *= 2) [29, 47], small noncoding RNA (*n *= 5) [37, 48, 50, 54, 56] and mRNA as a coding RNA (*n *= 1) [33] were the transcriptomic markers assessed by the included literature.

These tumor markers were detected by various detection techniques by the means of immunohistochemistry, molecular biology and biochemistry. Eighteen articles among the included articles have used different polymerase chain reaction (PCR) techniques to obtain accurate results [23, 24, 26, 29, 33, 34, 37, 38, 40, 41, 43, 47, 48, 50, 53, 54, 55, 56]. Also, several articles reported the use of immunohistochemical staining [35, 48], ELISA [27, 28, 30, 39, 42, 43, 45], colorimetric analysis [31, 44, 51, 52], and western blotting [25, 48] as the detection technique. One article each reported the use of Sanger sequencing [23], Tolbert's cytological examination [21], immunocytochemistry [46], cytometric bead assay [49], biochemical analysis [32], and bioinformatics [36]. Most of the articles also reported the use of biomarkers for detecting the risk of OSCC [22, 23, 24, 25, 26, 28, 30, 31, 34, 36, 37, 38, 43, 44, 48, 50, 52, 54, 55, 56]. Specific tumor markers were identified as early diagnosis tumor markers [21, 26, 27, 33, 41, 45, 46, 51], prognosis markers [29, 30, 35, 42, 45, 47, 49, 52, 53, 56], differentiation between OSCC and oral potentially malignant disorders (OPMDs) [22], oral dysplasia [32, 43], lymph node metastasis [40] and OSCC recurrence [43] (Table 2).

**Table 2:** Summary of the extracted findings.

Type of tumor marker	Tumor marker examined	Cancer sites	Sample type	Detection method	Key findings and conclusion	Study
1. Genomic markers	TP53 gene	Information not stated	Saliva sample Cancerous oral lesion tissues	PCR and Sanger sequencing	Codon 72 polymorphism and a heterozygous mutation at codon 172 were observed in TP53 gene of OSCC patients. Tissue samples which showed codon 72 polymorphism, matched salivary DNA sequence. Similarly, tissue samples which showed codon 172 polymorphism, matched salivary DNA sequence The ORR approach has the potential to be used in clinical settings as an alternative to invasive tissue biopsy to detect genetic alterations in assumed biomarkers in oral cancer.	D'Cruz et al. (2021) [23]
	Hypermethylated PAX1, ZNF582, SOX1, PTPRR, and NKX6.1	Oral mucosa, buccal mucosa	Oral scraping	Quantitative methylation‐specific PCR	Areca nut chewing, whether alone or combined with cigarette smoking or alcohol consumption, was observed to be associated with hypermethylation of ZNF582 and PAX1. Hypermethylated PAX1 and ZNF582 are effective biomarkers for detecting oral cancer and oral dysplasia, and for predicting the recurrence of oral cancer.	Cheng et al. (2016) [43]
	DNA methylated SOX1, PAX1 and ZNF582	Oral cavity cells (lateral tongue, alveolar ridge, floor of mouth, pharyngeal inlet, and buccal mucosa)	Oral swab	Quantitative methylation‐specific PCR	The methylation levels of PAX1, SOX1, and ZNF582 showed significant elevation in cancer patients. Patients exhibiting high methylation in SOX1, PAX1, and ZNF582 exhibited increased risk of cancer.	Huang et al. (2014) [55]
	Methylated ZNF582 and PAX1	Buccal, Tongue, Gingiva, Other sites	Oral epithelial cells	Quantitative methylation‐specific PCR	Patients with higher methylation levels of ZNF582 and PAX1 at baseline showed a higher risk of illness progression. ZNF582 hypermethylation may be a useful and noninvasive biomarker for diagnosing oral lesions with a high risk of malignancy.	Juan et al. (2023) [38]
	Promoter Hypermethylation of Tumor‐Suppressor Genes RASSF1A, p16INK4a, PCQAP/MED15, and TIMP3	Oral cavity [Tongue (Front 2/3), Hard palate, Buccal mucosa, Mouth floor, Retromolar area, Lips] Oropharynx [Tongue (Back 1/3), Soft palate, Tonsillar pillar, The back wall of the throat]	Saliva samples	MS‐PCR assay	Compared to healthy controls, TIMP3, PCQAP/MED15 TSGs, and RASSF1A were substantially hypermethylated in OC and OPC patients. Alcohol use, betel quid chewing, and smoking significantly increased TSG DNA methylation levels. The quadruple‐methylation marker panel of RASSF1A, p16INK4a, PCQAP/MED15 TSGs, and TIMP3 also revealed good diagnostic accuracy in the early diagnosis of OC and OPC in healthy individuals.	Liyanage et al. (2019) [41]
	GSTM1‐GSTT1 polymorphism and mtDNA	tumor tissue	Formalin‐fixed paraffin embedded tissue (FFPE) Tumor tissue Oral swab	Multiplex PCR and quantitative real‐time PCR	The likelihood of developing OSCC associated with a decrease in mtDNA copy numbers. Among tobacco–betel quid chewers, a significant association between OSCC risk and mtDNA copy number. Between the mtDNA content variation in cases and controls, a significant difference was observed between GSTT1 null genotypes, GSTM1, and HPV infection. A positive correlation was found between decreased mtDNA content and increased tumor stages.	Mondal et al. (2013) [53]
	Micronuclei	Oral exfoliated cells of buccal mucosa	Scrapings of buccal mucosa	Tolbert's cytological examination	A progressive rise in Micronuclei counts were indicated at potentially malignant and malignant stages. This suggested a probable association between Micronuclei with neoplastic progression. Therefore, Micronuclei can be used as a biomarker for early detection of oral premalignant lesions as well as oral malignant lesions.	Patel et al. (2021) [21]
	Chitinase‐3‐like protein 1 (CHI3L1) polymorphism e.g.: YKL‐40	Oral cavity	Whole blood specimens	Real‐time PCR	There was a notable link found between CHI3L1 polymorphism variants and the risk of oral cancer along with the risk factors such as smoking, betel nut chewing, and alcohol consumption. Patients with oral cancer who possess the homozygous A/A genotype of the CHI3L1 polymorphisms exhibited a notably reduced risk of lymph node metastasis. Furthermore, based on the Genotype‐Tissue Expression database, the CHI3L1 polymorphisms located in the promoter region were linked to reduced levels of CHI3L1 mRNA. Presence of a homozygous mutant allele of CHI3L1 polymorphisms significantly linked to a reduced risk of lymph node metastasis and was correlated with its mRNA levels in oral cancer. The CHI3L1 polymorphisms, could potentially serve as biomarkers to predict lymph node metastasis in individuals with oral cancer.	Su et al. (2019) [40]
	Promoter hyper methylation of LATS1 gene	Information not stated	Blood samples	Methylation‐specific PCR (MSP‐PCR)	Methylated LATS1 gene was statistically significant with smokers and tobacco chewers. This methylation pattern of the LATS1 gene was associated with an elevated likelihood of OSCC development. Consequently, the LATS1 gene emerges as a promising diagnostic biomarker.	Goel et al. (2021) [24]
2. Transcriptomic markers	lncRNAs: MALAT1, H19, CDKN2B‐AS1, HOTAIR, AP5M1, FALEC, LINC‐RoR, LINC00312, MEG3, POU3F3, PANDAR	Buccal mucosa, tongue, alveolar ridge, lip, and others	Tumor specimens Adjacent normal tissues	qRT‐PCR	Only MALAT1 HOTAIR, Linc‐RoR, CDKN2B‐AS1, AP5M1, FALEC, LINC00312, PANDAR, and POU3F3 were overexpressed in tumors associated with a history of tobacco chewing. Linc‐RoR was found to be significantly overexpressed in undifferentiated OSCC samples, and its overexpression was strongly associated with tumor recurrence and poor therapeutic response. The overexpression of linc‐RoR, coupled with the downregulation of miR‐145‐5p and the overexpression of Oct4, Klf4, c‐Myc, and Sox2 in OSCCs. Presence of a linc‐RoR‐mediated competing endogenous RNA network in undifferentiated tumors. There is an association of linc‐RoR overexpression in undifferentiated oral tumors and its prognostic significance in predicting therapeutic responses.	Arunkumar et al. (2017) [47]
	Circulating MIR‐21 and PTEN	oral squamous cell	Blood Samples	Quantitative RT‐PCR	Blood PTEN and MIR‐21 levels and were associated with the expression levels of tumor MIR21 and PTEN. Circulating MIR‐21 and PTEN could serve as new complementary tumor markers for OSCC.	Ren et al. (2014) [54]
	MIR‐21 and PTEN	Oral cavity	Blood Samples	qRT‐PCR	A noteworthy elevation observed in miR‐21 expression in OSCC compared to OSMF. Additionally, a positive connection was found between increased miR‐21 expression and individuals who chew pan‐masala. Also, a positive association noted between miR‐21 upregulation and advanced clinical stages of OSCC. However, additional studies are necessary to validate its potential as a reliable diagnostic and prognostic biomarker for OSMF and OSCC.	Singh et al. (2018) [56]
	MicroRNA (miR‐221‐3p, miR‐133a‐3p, and miR‐9‐5p)	Information not stated	Tissue and blood samples	qRT‐PCR	MiRNA133a‐3p levels were significantly lower in OSCC compared to OSMF and controls, but higher in OSMF compared to controls. There was no significant difference in miR‐221‐3p expression between OSCC and OSMF, although there was an increase in OSCC compared to controls. Both OSCC and OSMF were found to have increased levels of miR‐9‐5p. Furthermore, miR‐133a‐3p expression was linked with age, smoking, drinking status, and AJCC staging. MiR‐9‐5p expression was exclusively associated with tobacco/areca nut chewing. These markers can be employed as risk stratification biomarkers.	Ukey et al. (2023) [37]
	lncRNAs: UCA1, TUG1, HOTAIR, MALAT1, and H19	Lip, Tongue, Buccal Mucosa, Gingivobuccal sulcus, Alveoli, Hard Palate	Tumor and adjacent normal tissues	qRT‐PCR	The UCA1 and TUG1 genes showed upregulation in OSCC tumors, respectively. The expression of MALAT1 and H19 was observed to be downregulated in tumor tissues among OSCC patients. MALAT1, showed concordance with the TCGA analysis. HOTAIR expression in OSCC tumors had a positive correlation with tumor volume. A negative correlation was indicated by MALAT1 and H19 with the smokers.	Vishwakarma et al. (2020) [29]
	miR‐149 miR‐499 polymorphisms	Buccal mucosa, Tongue, Upper and Lower gingival	Blood samples	Quantitative real‐time PCR IHC Western blotting	The study suggests that miR‐499 A/G and miR‐149 C/T polymorphisms may play a significant role in both the susceptibility and development of OSCC.	Tandon et al. (2017) [48]
	MALAT1, H19, miR‐93‐5p, miR‐21‐5p, miR‐200c‐3p, miR‐205‐5p	Head, Tongue, Tonsils, Lip, Mandible/maxilla, Oral cavity, Gum, Palate	Fresh frozen tissue	Real‐Time Quantitative PCR	The findings underscore the significant involvement of H19, miR‐205‐5p, miR‐200c‐3p, miR‐93‐5p, and miR‐21‐5p in OSCC. Variations in their expression across different sub‐sites could potentially serve as diagnostic indicators, requiring further investigation with a larger sample size. Subsequent research should be conducted to confirm the interconnection with noncoding and coding genes. Top of FormBottom of Form	Aghiorghiesei et al. (2022) [50]
	mRNA levels of six genes NAB2, CYP27A1, NPIPB4, MAOB, SIAE, COL3A1	Information not stated	Saliva samples	Real‐Time PCR (qPCR)	Normalized mRNA levels of six genes were significantly lower in saliva of OSCC patients. Among all age groups the combination of CYP27A1 + SIAE yielded a favorable predictor for OSCC. Also, MAOB–NAB2 combination was more predictive of OSCC in the under‐60 years of age group. These findings are predicted to facilitate the early detection of OSCC, particularly among patients under the age of 60. Further studies with larger sample sizes are warranted; however, our results suggest that salivary mRNA could serve as a robust biomarker for early OSCC diagnosis.	Oh et al. (2020) [33]
3. Proteomic markers	Ceruloplasmin	Information not stated	Incisional biopsy: periphery of lesions and adjacent normal tissue	ELISA	Serum ceruloplasmin levels were higher in all oral leukoplakia, OSMF, and OSCC, as compared to controls, and the differences were statistically significant.	Patil et al. (2021) [28]
	MYO1B	Oral cancer tissue	Tissue chips	IHC	MYO1B overexpression has been linked to lymph node metastases and poor outcomes in oral cancer. MYO1B was found to have a positive correlation with macrophage, B cell, and dendritic cell infiltration. Suppression of MYO1B inhibit the ability of Arecoline‐transformed oral cells and oral cancer cells to proliferate, invade, and metastasize.	Sun et al. (2023) [35]
	CPLANE1	Tongue, Gingiva, Floor of the mouth, Buccal mucosa, multiple sites	Saliva samples	qRT‐PCR	CPLANE1 expression levels in OSCC patients were considerably greater than in HVs and OPMDs patients. Salivary CPLANE1 could serve as a valuable biomarker for screening and early detection of OSCC as it obtained accurate results.	Ueda et al. (2021) [26]
	CYFRA 21‐1	Oral mucosa	Saliva samples	ELISA	Expression of p53 among shisha smokers, cigarette smokers, and simultaneous smokers had statistical significance. However, there were no significance observed among the four study cohorts on CYFRA 21‐1 levels.	Amer et al. (2019) [39]
	Ki‐67 and MCM3	Information not mentioned	Oral brush biopsies	Immunocytochemistry (ICC)	Majority (96.4%) of OSCC cases contained MCM3 positive cells compared to Ki‐67 positive cells. All normal mucosa were Ki‐67 and MCM3 negative. In OSCC cases, both Ki‐67 and MCM3 labeling index (LI) were significantly elevated compared to normal mucosa. Moreover, MCM3 LI surpassed Ki‐67 LI within the OSCC cohort. These findings suggest that assessing Ki‐67 and MCM3 immunocytochemically could aid in the early identification of OSCC. Additionally, MCM3 serve as a more sensitive cytologic biomarker than Ki‐67 in patients with OSCC.	Rezazadeh et al. (2017) [46]
	Serum SNCG and SCCAg	Tongue, Buccal mucosa, Gingiva, Hard palate, Floor of mouth, Lip, Alveolar ridge	Blood sample	ELISA	Serum SNCG and SCCAg were significantly higher in OSCC compared with OPMDs. Combined approach of detecting SNCG and SCCAg in serum enhances the ability to differentiate between OSCC and OPMDs, thus elevating diagnostic accuracy for OSCC.	Chen et al. (2021) [22]
	Ki‑67 and Micronucleus	Buccal mucosa	buccal mucosal smear Saliva sample	ELISA	A notable contrast was observed in Ki‑67 levels and micronucleus counts between the betel nut chewers group and the control group. Moreover, significant disparities were noted in Ki‑67 and micronucleus levels across different types of oral lesions within the betel nut chewers group. These findings indicate that assessing Ki‑67 and micronucleus levels serves as an effective alternative for early detection of OSCC.	Triani et al. (2021) [27]
	Serum albumin, HSP (Heat shock protein) 27, gamma actin, SCC (Squamous cell carcinoma) 1, and Annexin A4.	Information not stated	Saliva samples	Western Blotting	Among Tamol chewers Serum Albumin, gamma actin, SSC 1, and ANX4 levels were elevated compared with healthy controls. Study suggests, salivary proteins have a positive association with OSCC development.	Lepcha et al. (2021) [25]
	CD4 + T cells expressing CD45RO and CCR6, regulatory T cells (T_reg_)	Gingiva Tongue Floor of the mouth Others (Cheek, Palate, Lip)	Tumor and gingival tissue Blood samples	Cytometric bead array (CBA)	OSCC patients with smoking and drinking habits exhibited increased levels of specific immune cells and cytokines compared to nonsmoking and non‐drinking patients and healthy donors, with implications for prognosis.	Rochefort et al., (2022) [49]
	Salivary and serum IL‐6	Buccal mucosa, Vestibule, Alveolar mucosa, Palate, Tongue, Lip	Saliva samples Blood samples	ELISA	Significant differences in IL‐6 levels were observed between OSCC and PML/C patients in both serum and saliva. In conclusion, our study indicates that the pro‐inflammatory cytokine IL‐6 is heightened in the saliva of OSCC patients compared to PMD individuals and controls. These findings suggest potential diagnostic or prognostic significance for IL‐6 in OSCC.	Dineshkumar et al. (2016) [45]
	TNF‐α	Buccal mucosa, Palate, Tongue, Floor of the mouth, Retromolar trigone, Alveolar mucosa, Oropharynx	Saliva samples	ELISA	Salivary TNF‐α concentrations exhibited notable elevation in individuals with OSCC compared to those with leukoplakia and healthy controls, with a high degree of statistical significance. Salivary TNF‐α emerges as a superior medium for OSCC detection. Moreover, its levels correlate with the severity of both OSCC and leukoplakia. This study concludes that salivary TNF‐α holds promise as a prognostic biomarker for OSCC. Considering the heightened TNF‐α levels in the saliva of individuals with severe dysplasia, it also presents potential for monitoring the malignant progression from leukoplakia to OSCC.	Deepthi et al. (2019) [42]
4. Metabolomic markers	Salivary sialic acid	Information not stated	Saliva samples	Colorimetric analysis	Salivary PBSA and FSA levels are considerably higher in both tobacco chewers with OSCC and in tobacco chewers with no precancerous or cancerous lesions in the oral cavity.	Azeem et al. (2020) [31]
	Salivary lactate Dehydrogenase	Oral squamous cells	Saliva samples	Colorimetric analysis	Elevated levels of salivary LDH were observed in patients with Leukoplakia and OSCC. However, smoking alone did not cause any changes in salivary LDH. This suggests that salivary LDH might serve as a potential biomarker for detecting early premalignant or malignant changes in smokers.	Bhuvaneswari et al. (2022) [51]
	Serum glycoconjugates: Protein bound hexoses, Sialic acid Fucose	Information not stated	Blood samples	Colorimetric analysis	Serum glycoconjugate levels were high in OLK, OSF, and Oral cancer groups compared to tobacco and nontobacco controls. Serum glycoconjugates can be used as a diagnostic and prognostic marker in OLK, OSF and in Oral cancer.	Bose et al. (2013) [52]
	Salivary sialic acid	Information not stated	Saliva samples	Colorimetric analysis	Salivary sialic acid levels were high in OSCC compared to OLK, Erythroplakia, smokers’ palate, smokeless tobacco keratosis and control group. Statistical significance was observed among OSCC stages, dysplasia stages in premalignancies and sialic acid level. Salivary sialic acid levels in OSCC, OLK, Erythroplakia smokers’ palate and smokeless tobacco keratosis can be used as a diagnostic tool.	Chaudhari et al. (2016) [44]
	Salivary MMP‐9	Alveolus (mandible, maxilla, maxillary), Buccal mucosa, Floor of the mouth, Hard palate, Lateral border of tongue, Retromolar trigone, Lip	Saliva sample	ELISA	Individuals diagnosed with OSCC and OPMD indicated elevated mean MMP‐9 levels compared to those with tobacco habits and control groups. Additionally, the poorly differentiated OSCC subgroup demonstrated notably higher mean saliva MMP‐9 levels in comparison to the moderate and well‐differentiated OSCC subgroups. Salivary MMP‐9 can be used as an noninvasive alternative marker in diagnosing, treating and monitoring OSCC and OPMD.	Smriti et al. (2020) [30]
	Serum Alkaline Phosphates Lactate Dehydrogenase	Information not stated	Saliva sample Blood sample	Biochemical analysis	This study revealed that there was high expression of both serum and salivary ALP and LDH in patients with OPMDs and OSCC compared to other cohorts. Serum and salivary ALP was high among individuals with OPMD who consume tobacco. Both ALP and LDH could be deemed sensitive markers for detecting dysplasia in the presence of pre‐cancerous and cancerous lesions.	Goyal (2019) [32]
5. Microbiome	*Prevotella*, *Fusobacterum*, *Veillonella*, *Haemophilus*, *Capnocytophaga*, *Leptotrichia*	Oral cavity	Oral swab sample	qRT‑PCR	The use of smokeless tobacco (SLT) and the development of oral premalignant lesions (OPL) are linked to an imbalance in the oral bacteriome, suggesting an increase in bacterial species known to play a role in oral cancer development.	Sajid et al. (2023) [34]
	*Pseudomonas*, *Mycoplasma*, *Capnocytophaga*	Information not stated	Oral rinse samples	Bioinformatics	*Pseudomonas*, *Capnocytophaga*, and *Mycoplasma* were highly abundant in tobacco chewers, whereas *Pseudomonas*, *Capnocytophaga*, and *Mycoplasma* were significantly enriched in oral cancer. In the oral cancer group, genes involved in lipid biosynthesis and fatty acid elongation were elevated, whereas those involved in the reductive TCA cycle were upregulated in the tobacco group.	Sawant et al, (2023) [36]

Abbreviations: AJCC, American Joint Committee on Cancer; CHI3L1, Chitinase 3 like 1; c‐Myc, cellular myelocytomatosis oncogene; CPLANE1, ciliogenesis and planar polarity effector complex subunit 1; CYFRA 21‐1, cytokeratin 19 fragment; SNCG, synuclein‐γ; FSA, free sialic acid; GSTM1, glutathione S‐transferase mu 1; GSTT1, glutathione *S*‐transferase theta 1; IL‐6, Interleukin 6; Ki‐ 67, Antigen Kiel 67; Klf4, Krüppel‐like factor 4; LDH, Lactate dehydrogenase; LINC‐ROR, long intergenic non‐protein coding RNA, regulator of reprogramming; MALAT1, Metastasis Associated Lung Adenocarcinoma Transcript 1; MCM3, Minichromosome Maintenance Complex Component 3; miR‐9‐5p, microRNA‐9‐5p; miR‐145‐5p, micro RNA 145‐5p; miR‐221‐3p, microRNA‐221‐3p; MiRNA133a‐3p, microRNA133a‐3p; MIR21, microRNA 21; Oct4; Octamer Transcription Factor 4; mtDNA, mitochondrial DNA; MYO1B, Myosin 1B; OC, oral cancer; OPC, oropharyngeal cancer; ORR, oral rub and rinse; p16INK4a, inhibitor of cyclin‐dependent kinase 4a; PAX1m, methylated paired box 1; PBSA, protein‐bound sialic acid; PCQAP/MED15, Mediator complex subunit 15; PTEN, phosphatase and tensin homolog; RASSF1, Ras association domain family member 1; SCCAg, squamous cell carcinoma antigen; SOX1, sex determining region Y box 1; TIMP 3, metallopeptidase inhibitor 3; TNF‐α, Tumor Necrosis Factor‐α; TSG, tumor suppressor genes; TUG1, taurine upregulated 1; UCA1, urothelial carcinoma associated 1; ZNF582m, methylated zinc finger protein 582.

## 4 Discussion

At present, the search for noninvasive, cost‐effective, and accurate diagnosis methods for OSCC is in demand, yet there was no significant reduction in mortality rates globally [17]. Therefore, identifying the best suitable early diagnosis tumor marker of OSCC assists in disease management by diminishing prevalence and the mortality rates. Thereby, this scoping review aimed to provide an overview of genomic markers, transcriptomic markers, proteomic markers, metabolomic markers, and microbiome dysbiosis which contributed to early diagnosis of OSCC induced by tobacco and betel quid. Of all the selected literature, the tumor markers explicitly indicated with different populations, study cohorts, study designs and detecting techniques.

The bibliometric analysis of the characteristics of the included literature revealed an increase in research on diagnostic tumor markers in tobacco‐ and betel quid‐induced OSCC within the past 5 years. Considering the geographical locations of the studies, it was mostly limited to the Asian continent with India being the country with the most publications on diagnostic tumor markers in tobacco‐ and betel quid‐induced OSCC. However, there was no publication reported from North America, South America and Australia, according to this scoping review's findings. Also, the study cohorts of the included literature were between 15 and 97 years of age. In almost all the studies, the efficacy of the tumor markers was assessed on both genders, except for the study which was conducted only in the male population of Taiwan [40].

In this scoping review, the studies which assessed on DNA polymorphisms, codon 72 and 172 polymorphisms of TP53 gene [23] and homozygous mutant allele of CHI3L1 polymorphism [40] were independent predictors in OSCC [23, 40] and lymph node metastasis of OSCC [40] respectively. However, GSTM1‐GSTT‐1 polymorphisms depended on the mtDNA copy number in assessing the tumor stages of OSCC as well as risk of OSCC [53]. Methylated *ZNF582, PAX1*, and *SOX1* were the most assessed DNA methylation in included studies [38, 43, 55]. Exposure to risk habits such as betel chewing, smoking and alcohol consumption independently or in combined forms will lead to hypermethylation of ZNF582, PAX1 and SOX1 genes and tumor suppressor genes [41, 43].

Among the studies which assessed on lncRNAs of transcriptomic markers, only *CDKN2B‐AS1*, *HOTAIR*, *AP5M1*, *FALEC*, *LINC00312*, *linc‐RoR*, *PANDAR*, *POU3F3*, *MALAT1* [47] *UCA1* and *TUG1* [29] were overexpressed in tumors. However, contrary findings were reported by Vishwakarma *et al*. (2020) [29], where *MALAT1* and *H19* were negatively correlated with smoking status [29]. *LINC‐ROR* was specifically identified as a prognostic marker in OSCC due to its statistical significance in undifferentiated OSCC, history of tobacco chewing or smoking, therapeutic response and tumor recurrence [47]. Among the sncRNAs, MIR 21 was the most assessed tumor marker, and it was assessed together with PTEN. Both blood MIR 21 and PTEN were upregulated in OSCC patients [54, 56]. However, when considering MIR 21 independently, it was upregulated in individuals exposed to risk habits, particularly pan masala [56]. Similarly, the expression of miR‐9‐5p in both tissue and blood were exclusively associated with the risk factor areca nut chewing [37]. In contrast, the dysregulated expressions of miR‐21‐5p, miR‐93‐5p, miR‐200c‐3p, and miR‐205‐5p, along with risk habits such as smoking, did not show any correlation [50]. Additionally, *NAB2*, *CYP27A1*, *NPIPB4*, *MAOB*, *SIAE*, *COL3A1* gene alterations at mRNA level were downregulated in OSCC [33]. Nevertheless, combination of genes was more efficient in early diagnosis of OSCC, compared to independent mRNA level gene alterations. Also, the combination of *MAOB–NAB2* was identified as an age‐specific tumor marker [33].

Among the proteomic markers, elevated levels of serum ceruloplasmin [28], MYO1B [35], salivary *CPLANE1* [26], serum albumin, HSP 27, gamma actin, SCC 1, and A4 [25], serum SNCG and SCCAg [22] and immune cell markers such as, IL‐6 [45], TNF‐α [42], and CD4 + T cell [49] were suitable proteomic tumor markers in detecting OSCC. However, contradictory findings were obtained for salivary *CYFRA 21‐1* expression levels as there was no statistical significance reported among shisha smokers, cigarette smokers and simultaneous smokers [39]. Furthermore, two studies examined on the efficacy of Ki‐67 markers in comparison to MCM3 [46] and micronucleus [27], yielding conflicting results. According to Rezazadeh *et al*. (2017) [46], only MCM3 was suggested as a suitable marker for OSCC detection compared to Ki‐67. However, according to Triani et al. (2021) [27], both Ki‐67 and micronuclei can be used as an early diagnosis marker for OSCC as they are elevated due to exposure of betel nut chewing and severity of the lesion.

Salivary and serum sialic acid was the most assessed metabolomic tumor marker among the articles reviewed [31, 44, 52]. Protein‐bound and free form of salivary sialic acid were significantly higher in individuals who are exposed to smokeless tobacco [31]. Also, salivary and serum sialic acid levels were high in OSCC, OSF and OLK compared to erythroplakia, smokers’ palate, and smokeless tobacco keratosis [44, 52]. Apart from that, protein bound hexoses were significantly high in OLK, OSF, and oral cancer groups with or without tobacco chewing [52]. The efficacy of salivary LDH as a tumor marker in OSCC was independently assessed in only one study. Based on its findings, elevated salivary LDH levels were observed in OLK and OSCC, thus the upregulation was independent of the risk of smoking [51]. Moreover, only one study assessed on the efficacy of salivary and blood ALP and LDH. Thereby, it was proposed that salivary ALP and LDH were more effective OSCC tumor marker compared to serum ALP and LDH [32].

According to the included literature, only two studies assessed the tobacco‐induced dysbiosis of the oral microbiome as a OSCC tumor marker [34, 36]. Among the smokeless tobacco users with oral lesions, *Prevotella*, *Fusobacterum*, *Veillonella*, *Haemophilus*, *Capnocytophaga* and *Leptotrichia* of the oral bacteriome were overexpressed leading to an imbalance [34]. Likewise, compositional alterations of *Pseudomonas*, *Capnocytophaga*, and *Mycoplasma* were high in tobacco chewers [36]. Additionally, microbiome dysbiosis was significantly high in OSCC compared to premalignant stages. Thereby, oral microbiome is a suitable tumor marker to assess transition of OPMDs to OSCC [34, 36].

### 4.1 Limitations and Strengths of the Study

There were several limitations of this scoping review. The key limitation was all the included literature were restricted to English language. This might have excluded relevant studies in other languages, potentially leading to inclusion bias based on language restriction. Another limitation of this review was the heterogeneity of the study designs, study cohorts and methodologies. This heterogeneity limits the opportunity to compare and generalize the findings of this scoping review. Additionally, a significant proportion of the studies were conducted in Asia, particularly in India. This limits the applicability of the findings to other geographical regions with different risk factors and populations. Furthermore, further studies of a larger sample size is required to validate the tumor markers reported in the included studies, as some of the reported findings in the reviewed were contradictory [22, 23, 24, 27, 29, 31, 37, 40, 44, 45, 46, 47, 50, 54, 56].

Despite the limitations, this scoping review has its own strengths. The main strength was this scoping review was able to cover a thorough and systematic search of seven major databases, to obtain relevant studies on diagnostic tumor markers for OSCC. The rigorous screening and selection process ensured that a wide range of tumor markers were identified, including micronuclei frequency, miRNAs, protein markers, gene alterations, and salivary markers; hence, providing a comprehensive overview of potential diagnostic tools for OSCC. Furthermore, this scoping review highlighted existing gaps in the empirical evidence, guiding future research directions in the field of OSCC diagnostics.

## 5 Conclusion

This scoping review has contributed to the growing body of knowledge surrounding diagnostic markers in OSCC. Within the limitations, this scoping review was able to provide valuable insights into potential avenues for improving early detection and management of tobacco‐ and betel quid‐induced OSCC via noninvasive methods. However, large‐scale cohorts are required in validating these tumor markers.

## Author Contributions

**Yovanthi Anurangi Jayasinghe:** conceptualization, investigation, writing – original draft, methodology, validation, visualization, writing – review and editing, software, formal analysis, data curation, resources. **Kalpani Senevirathna:** investigation, writing – original draft, methodology, validation, visualization, writing – review and editing, software, formal analysis, resources, data curation. **Afeez Abolarinwa Salami:** writing – original draft, methodology, writing – review and editing, validation, visualization, resources. **Kehinde Kazeem Kanmodi:** investigation, funding acquisition, methodology, writing – original draft, writing – review and editing, validation, software, project administration, supervision, resources, visualization. **Ruwan Duminda Jayasinghe:** resources, supervision, project administration, writing – review and editing, validation, funding acquisition, investigation, conceptualization, methodology, software.

## Ethics Statement

The authors have nothing to report.

## Conflicts of Interest

Kehinde Kazeem Kanmodi and Ruwan Duminda Jayasinghe are Editorial Board members of *Health Science Reports* and co‐authors of this article. To minimize bias, they were excluded from all editorial decision‐making related to the acceptance of this article for publication. Other authors declare no conflicts of interest.

## Transparency Statement

The lead author Kehinde Kazeem Kanmodi affirms that this manuscript is an honest, accurate, and transparent account of the study being reported; that no important aspects of the study have been omitted; and that any discrepancies from the study as planned (and, if relevant, registered) have been explained.

## Supporting information

Supporting file Revised.

## Data Availability

The authors confirm that the data supporting the findings of this study are available within the article and its supporting materials.
