# Supplementary material for: The Use of Diagnostic Tumor Markers in Detecting Tobacco‐ and Betel Quid‐Induced Oral Squamous Cell Carcinoma: A Scoping Review of Empirical Evidence
Source: Health Sci Rep. 2025 Apr 18;8(4):e70650. doi: 10.1002/hsr2.70650 (PMC12007468; doi:10.1002/hsr2.70650)
Supplement: Supplementary file 1 — Supporting file Revised. [file HSR2-8-e70650-s001.docx]

**SUPPLEMENTARY FILE**

**Table S1. Search strings used for the PubMed database search.**

| **Tag** | **To search for:** | **Search strings** |
| --- | --- | --- |
| #1 | Diagnostic tumor markers | (((Diagnostic tumor marker[Title/Abstract]) OR (tumor marker[Title/Abstract])) OR (biomarker[Title/Abstract])) OR (biological marker[Title/Abstract]) |
| #2 | Detection | (((detect*[Title/Abstract]) OR (diagnos*[Title/Abstract])) OR (identif*[Title/Abstract])) OR (screen*[Title/Abstract]) |
| #3 | Tobacco/betel quid | ((((((((((tobacco[Title/Abstract]) OR (smoking[Title/Abstract])) OR (cigarette[Title/Abstract])) OR (shisha[Title/Abstract])) OR (waterpipe[Title/Abstract])) OR (hubble bubble[Title/Abstract])) OR (narghile[Title/Abstract])) OR (cigar[Title/Abstract])) OR (betel[Title/Abstract])) OR (quid[Title/Abstract])) OR (areca[Title/Abstract]) |
| #4 | Oral squamous cell carcinoma | ((((((((oral cancer[Title/Abstract]) OR (oral cavity cancer[Title/Abstract])) OR (lip cancer[Title/Abstract])) OR (cancer of the lip[Title/Abstract])) OR (tongue cancer[Title/Abstract])) OR (cancer of the tongue[Title/Abstract])) OR (oral squamous cell carcinoma[Title/Abstract])) OR (oral carcinoma[Title/Abstract])) OR (intra-alveolar squamous cell carcinoma[Title/Abstract]) |
| #5 | #1 AND #2 AND #3 AND #4 | (((#1) AND (#2)) AND (#3)) AND (#4) |

**Table S2. Search strings used for the SCOPUS database search.**

| **Tag** | **To search for:** | **Search strings** |
| --- | --- | --- |
| #1 | Diagnostic tumor markers | ( TITLE-ABS-KEY ( "diagnostic tumor marker" )  OR  TITLE-ABS-KEY ( "tumor marker" )  OR  TITLE-ABS-KEY ( biomarker )  OR  TITLE-ABS-KEY ( "biological marker" ) ) |
| #2 | Detection | ( TITLE-ABS-KEY ( detect* )  OR  TITLE-ABS-KEY ( diagnos* )  OR  TITLE-ABS-KEY ( identif* )  OR  TITLE-ABS-KEY ( screen* ) ) |
| #3 | Tobacco/betel quid | ( TITLE-ABS-KEY ( tobacco )  OR  TITLE-ABS-KEY ( smoking )  OR  TITLE-ABS-KEY ( cigarette )  OR  TITLE-ABS-KEY ( shisha )  OR  TITLE-ABS-KEY ( waterpipe )  OR  TITLE-ABS-KEY ( "hubble bubble" )  OR  TITLE-ABS-KEY ( narghile )  OR  TITLE-ABS-KEY ( cigar )  OR  TITLE-ABS-KEY ( betel )  OR  TITLE-ABS-KEY ( quid )  OR  TITLE-ABS-KEY ( areca ) ) |
| #4 | Oral squamous cell carcinoma | ( TITLE-ABS-KEY ( "oral cancer" )  OR  TITLE-ABS-KEY ( "oral cavity cancer" )  OR  TITLE-ABS-KEY ( "lip cancer" )  OR  TITLE-ABS-KEY ( "cancer of the lip" )  OR  TITLE-ABS-KEY ( "tongue cancer" )  OR  TITLE-ABS-KEY ( "cancer of the tongue" )  OR  TITLE-ABS-KEY ( "oral squamous cell carcinoma" )  OR  TITLE-ABS-KEY ( "oral carcinoma" )  OR  TITLE-ABS-KEY ( "intra-alveolar squamous cell carcinoma" ) ) |
| #5 | #1 AND #2 AND #3 AND #4 | ( ( TITLE-ABS-KEY ( "diagnostic tumor marker" ) OR TITLE-ABS-KEY ( "tumor marker" ) OR TITLE-ABS-KEY ( biomarker ) OR TITLE-ABS-KEY ( "biological marker" ) ) ) AND ( ( TITLE-ABS-KEY ( detect* ) OR TITLE-ABS-KEY ( diagnos* ) OR TITLE-ABS-KEY ( identif* ) OR TITLE-ABS-KEY ( screen* ) ) ) AND ( ( TITLE-ABS-KEY ( tobacco ) OR TITLE-ABS-KEY ( smoking ) OR TITLE-ABS-KEY ( cigarette ) OR TITLE-ABS-KEY ( shisha ) OR TITLE-ABS-KEY ( waterpipe ) OR TITLE-ABS-KEY ( "hubble bubble" ) OR TITLE-ABS-KEY ( narghile ) OR TITLE-ABS-KEY ( cigar ) OR TITLE-ABS-KEY ( betel ) OR TITLE-ABS-KEY ( quid ) OR TITLE-ABS-KEY ( areca ) ) ) AND ( ( TITLE-ABS-KEY ( "oral cancer" ) OR TITLE-ABS-KEY ( "oral cavity cancer" ) OR TITLE-ABS-KEY ( "lip cancer" ) OR TITLE-ABS-KEY ( "cancer of the lip" ) OR TITLE-ABS-KEY ( "tongue cancer" ) OR TITLE-ABS-KEY ( "cancer of the tongue" ) OR TITLE-ABS-KEY ( "oral squamous cell carcinoma" ) OR TITLE-ABS-KEY ( "oral carcinoma" ) OR TITLE-ABS-KEY ( "intra-alveolar squamous cell carcinoma" ) ) ) |

**Table S3. Search strings used for the search on other databases (Allied and Complementary Medicine Database (AMED), CINAHL Ultimate, APA PsycArticles, APA PsycINFO, and Dentistry and Oral Sciences Source) via the EBSCOHost Interface.**

| **Tag** | **To search for:** | **Search strings** |
| --- | --- | --- |
| S1 | Diagnostic tumor markers | AB diagnostic tumor marker OR AB tumor marker OR AB biomarker OR AB biological marker |
| S2 | Detection | AB detect* OR AB diagnos* OR AB identif* OR AB screen* |
| S3 | Tobacco/betel quid | AB tobacco OR AB smoking OR AB cigarette OR AB shisha OR AB waterpipe OR AB hubble bubble OR AB narghile OR AB cigar OR AB betel OR AB quid OR AB areca |
| S4 | Oral squamous cell carcinoma | AB oral cancer OR AB oral cavity cancer OR AB lip cancer OR AB cancer of the lip OR AB tongue cancer OR AB cancer of the tongue OR AB oral squamous cell carcinoma OR AB oral carcinoma OR AB intra-alveolar squamous cell carcinoma |
| S5 | S1 AND S2 AND S3 AND S4 | S1 AND S2 AND S3 AND S4 |

**Table S4. List of the literature which were considered for full text evaluation.**

| **No.** | **Citations** | **Included** | **Excluded (with Reasons)** |
| --- | --- | --- | --- |
| 1 | Patel TS, Chaudhary AR, Dudhia BB, Bhatia PV, Patel PS, Jani YV. A study on micronuclei in tobacco and related habits. J Indian Acad Oral Med Radiol. 2021;33(2):163-70. | Yes |  |
| 2 | Woo S, Gao H, Henderson D, Zacharias W, Liu G, Tran QT, Prasad GL. AKR1C1 as a Biomarker for Differentiating the Biological Effects of Combustible from Non-Combustible Tobacco Products. Genes (Basel). 2017 May 3;8(5):132. doi: 10.3390/genes8050132. PMID: 28467356; PMCID: PMC5448006. |  | Yes (Wrong study design) |
| 3 | Sajid M, Sharma P, Srivastava S, Hariprasad R, Singh H, Bharadwaj M. Alteration of oral bacteriome of smokeless tobacco users and their association with oral cancer. Appl Microbiol Biotechnol. 2023 Jun;107(12):4009-4024. doi: 10.1007/s00253-023-12534-z. Epub 2023 May 8. PMID: 37154908. | Yes |  |
| 4 | Padma R, Sundaresan S, Kalaivani A, Thilagavathi R. Assessment of Histopathological Grade and Ki-67 Expression in Tobacco and Non-tobacco Habitual Buccal Mucosa Cancer. Indian J Otolaryngol Head Neck Surg. 2019 Oct;71(Suppl 1):410-416. doi: 10.1007/s12070-018-1328-1. Epub 2018 Apr 6. PMID: 31741996; PMCID: PMC6848396. |  | Yes (Wrong study objectives) |
| 5 | Chen L, Luo T, Yang J, Wang K, Liu S, Wei Y, Liu H, Xu J, Zheng J, Zeng Y. Assessment of serum synuclein-γ and squamous cell carcinoma antigen as diagnostic biomarkers in patients with oral squamous cell carcinoma and oral potentially malignant disorders. J Oral Pathol Med. 2021 Feb;50(2):165-174. doi: 10.1111/jop.13115. Epub 2020 Oct 29. PMID: 33064859. | Yes |  |
| 6 | Shriddha A, Narain SA, Gul MA, Shariq IM, Ausaf A. Association of cancer stem cell markers CD44 and CD133 expression with clinicopathological changes in Oral squamous cell carcinoma (OSCC) and oral submucosal fibrosis (OSMF). Res J Biotechnol 2023;18(1);84-93. |  | Yes (Wrong study objectives) |
| 7 | Mondal R, Ghosh SK, Talukdar FR, Laskar RS. Association of mitochondrial D-loop mutations with GSTM1 and GSTT1 polymorphisms in oral carcinoma: a case control study from northeast India. Oral Oncol. 2013 Apr;49(4):345-53. doi: 10.1016/j.oraloncology.2012.11.003. Epub 2012 Dec 20. PMID: 23265943. |  | Full article not found |
| 8 | Singh P, Srivastava AN, Sharma R, Mateen S, Shukla B, Singh A, Chandel S. Circulating MicroRNA-21 Expression as a Novel Serum Biomarker for Oral Sub-Mucous Fibrosis and Oral Squamous Cell Carcinoma. Asian Pac J Cancer Prev. 2018 Apr 27;19(4):1053-1057. doi: 10.22034/APJCP.2018.19.4.1053. PMID: 29699056; PMCID: PMC6031776. | Yes |  |
| 9 | Ren W, Qiang C, Gao L, Li SM, Zhang LM, Wang XL, Dong JW, Chen C, Liu CY, Zhi KQ. Circulating microRNA-21 (MIR-21) and phosphatase and tensin homolog (PTEN) are promising novel biomarkers for detection of oral squamous cell carcinoma. Biomarkers. 2014;19(7):590-6. | Yes |  |
| 10 | Rochefort J, Karagiannidis I, Baillou C, Belin L, Guillot-Delost M, Macedo R, Le Moignic A, Mateo V, Soussan P, Brocheriou I, Teillaud JL, Dieu-Nosjean MC, Bertolus C, Lemoine FM, Lescaille G. Defining biomarkers in oral cancer according to smoking and drinking status. Front Oncol. 2023 Jan 11;12:1068979. doi: 10.3389/fonc.2022.1068979. PMID: 36713516; PMCID: PMC9875375. |  | Yes (Wrong study population) |
| 11 | Khyani IAM, Qureshi MA, Mirza T, Farooq MU. Detection of interleukins-6 and 8 in saliva as potential biomarkers of oral pre-malignant lesion and oral carcinoma: A breakthrough in salivary diagnostics in Pakistan. Pak J Pharm Sci. 2017 May;30(3):817-823. PMID: 28653927. |  | Yes (Wrong study design) |
| 12 | Ramesh B, Aswath N, Shyamsundar VR. Determining the probability of malignant transformation of tobacco-induced oral leukoplakia using tissue p53 as a prognostic marker – A cross-sectional study. J Indian Acad Oral Med Radiol. 2022;34(2):126-30. |  | Yes (Wrong study design) |
| 13 | Tseng YJ, Wang YC, Hsueh PC, Wu CC. Development and validation of machine learning-based risk prediction models of oral squamous cell carcinoma using salivary autoantibody biomarkers. BMC Oral Health. 2022 Nov 24;22(1):534. doi: 10.1186/s12903-022-02607-2. PMID: 36424594; PMCID: PMC9685866. |  | Yes (Wrong study design) |
| 14 | Amer HW, Waguih HM, El-Rouby DH. Development of field cancerization in the clinically normal oral mucosa of shisha smokers. Int J Dent Hyg. 2019 Feb;17(1):39-45. doi: 10.1111/idh.12362. Epub 2018 Sep 21. PMID: 30113759. | Yes |  |
| 15 | Huang YK, Peng BY, Wu CY, Su CT, Wang HC, Lai HC. DNA methylation of PAX1 as a biomarker for oral squamous cell carcinoma. Clin Oral Investig. 2014 Apr;18(3):801-8. doi: 10.1007/s00784-013-1048-6. Epub 2013 Aug 2. PMID: 23907469. | Yes |  |
| 16 | Aghiorghiesei O, Zanoaga O, Raduly L, Aghiorghiesei AI, Chiroi P, Trif A, Buiga R, Budisan L, Lucaciu O, Pop LA, Braicu C, Campian R, Berindan-Neagoe I. Dysregulation of miR-21-5p, miR-93-5p, miR-200c-3p and miR-205-5p in Oral Squamous Cell Carcinoma: A Potential Biomarkers Panel? Curr Issues Mol Biol. 2022 Apr 16;44(4):1754-1767. doi: 10.3390/cimb44040121. PMID: 35723379; PMCID: PMC9164081. | Yes |  |
| 17 | Bhuvaneswari M, Prasad H, Rajmohan M, Sri Chinthu KK, Prema P, Mahalakshmi L, Kumar GS. Estimation of salivary lactate dehydrogenase in oral squamous cell carcinoma, oral leukoplakia, and smokers. J Cancer Res Ther. 2022 Dec;18(Supplement):S215-S218. doi: 10.4103/jcrt.JCRT_969_20. PMID: 36510967. | Yes |  |
| 18 | Rezazadeh F, Ebrahimi R, Andisheh-Tadbir A, Ashraf MJ, Khademi B. Evaluation of the Ki-67 and MCM3 Expression in Cytologic Smear of Oral Squamous Cell Carcinoma. J Dent (Shiraz). 2017 Sep;18(3):207-211. PMID: 29034276; PMCID: PMC5634361. | Yes |  |
| 19 | Vishwakarma S, Pandey R, Singh R, Gothalwal R, Kumar A. Expression of H19 long non-coding RNA is down-regulated in oral squamous cell carcinoma. J Biosci. 2020;45:145. PMID: 33410422. | Yes |  |
| 20 | Arunkumar G, Deva Magendhra Rao AK, Manikandan M, Arun K, Vinothkumar V, Revathidevi S, Rajkumar KS, Rajaraman R, Munirajan AK. Expression profiling of long non-coding RNA identifies linc-RoR as a prognostic biomarker in oral cancer. Tumour Biol. 2017 Apr;39(4):1010428317698366. doi: 10.1177/1010428317698366. PMID: 28443494. | Yes |  |
| 21 | Su CW, Chen MK, Hung WC, Yang SF, Chuang CY, Lin CW. Functional variant of CHI3L1 gene is associated with neck metastasis in oral cancer. Clin Oral Investig. 2019 Jun;23(6):2685-2694. doi: 10.1007/s00784-018-2683-8. Epub 2018 Oct 19. PMID: 30341592. | Yes |  |
| 22 | Cheng SJ, Chang CF, Lee JJ, Chen HM, Wang HJ, Liou YL, Yen C, Chiang CP. Hypermethylated ZNF582 and PAX1 are effective biomarkers for detection of oral dysplasia and oral cancer. Oral Oncol. 2016 Nov;62:34-43. doi: 10.1016/j.oraloncology.2016.09.007. Epub 2016 Oct 6. PMID: 27865370. | Yes |  |
| 23 | Sarkar R, Chatterjee K, Ojha D, Chakraborty B, Sengupta S, Chattopadhyay D, RoyChaudhuri C, Barui A. Liaison between heme metabolism and bioenergetics pathways-a multimodal elucidation for early diagnosis of oral cancer. Photodiagnosis Photodynamic Therapy. 2018;21:263-74. |  | Yes (Wrong study design) |
| 24 | Tandon D, Dewangan J, Srivastava S, Garg VK, Rath SK. miRNA genetic variants: As potential diagnostic biomarkers for oral cancer. Pathol Res Pract. 2018 Feb;214(2):281-289. doi: 10.1016/j.prp.2017.10.002. Epub 2017 Oct 10. PMID: 29103762. | Yes |  |
| 25 | Mondal R, Ghosh SK, Choudhury JH, Seram A, Sinha K, Hussain M, Laskar RS, Rabha B, Dey P, Ganguli S, Nathchoudhury M, Talukdar FR, Chaudhuri B, Dhar B. Mitochondrial DNA copy number and risk of oral cancer: a report from Northeast India. PLoS One. 2013;8(3):e57771. doi: 10.1371/journal.pone.0057771. Epub 2013 Mar 4. PMID: 23469236; PMCID: PMC3587625. | Yes |  |
| 26 | Sun Z, Guo X, Chen H, Ling J, Zhao H, Chang A, Zhuo X. MYO1B as a prognostic biomarker and a therapeutic target in Arecoline-associated oral carcinoma. Mol Carcinog. 2023 Jul;62(7):920-939. doi: 10.1002/mc.23535. Epub 2023 Apr 4. PMID: 37014156. | Yes |  |
| 27 | D'Cruz A, Dechamma PN, Saldanha M, Maben S, Shetty P, Chakraborty A. Non-Invasive Saliva-based Detection of Gene Mutations in Oral Cancer Patients by Oral Rub and Rinse Technique. Asian Pac J Cancer Prev. 2021 Oct 1;22(10):3287-3291. doi: 10.31557/APJCP.2021.22.10.3287. PMID: 34711005; PMCID: PMC8858228. | Yes |  |
| 28 | Sawant S, Dugad J, Parikh D, Srinivasan S, Singh H. Oral Microbial Signatures of Tobacco Chewers and Oral Cancer Patients in India. Pathogens. 2023 Jan 3;12(1):78. doi: 10.3390/pathogens12010078. PMID: 36678424; PMCID: PMC9864012. | Yes |  |
| 29 | Li C, Zhou Y, Liu J, Su X, Qin H, Huang S, Huang X, Zhou N. Potential Markers from Serum-Purified Exosomes for Detecting Oral Squamous Cell Carcinoma Metastasis. Cancer Epidemiol Biomarkers Prev. 2019 Oct;28(10):1668-1681. doi: 10.1158/1055-9965.EPI-18-1122. Epub 2019 Jul 26. PMID: 31350263. |  | Yes (Wrong study design) |
| 30 | Gouvea SA, de Podesta JR, de Freitas Cordeiro-Silva M, Louro ID, Von Zeidler SV. PP078: Epigenetic silencing of cancer-related genes in tumors and non-neoplastic cells from patients with oral squamous cell carcinoma. Oral Oncol. 2013;49:S120-1. |  | Yes (Wrong publication type) |
| 31 | Goel H, Mathur R, Syeda S, Shrivastava A, Jha AK. Promoter Hypermethylation of LATS1 Gene in Oral Squamous Cell Carcinoma (OSCC) among North Indian Population. Asian Pac J Cancer Prev. 2021 Mar 1;22(3):977-982. doi: 10.31557/APJCP.2021.22.3.977. PMID: 33773564; PMCID: PMC8286665. | Yes |  |
| 32 | Liyanage C, Wathupola A, Muraleetharan S, Perera K, Punyadeera C, Udagama P. Promoter Hypermethylation of Tumor-Suppressor Genes *p16*^INK4a^*,RASSF1A,TIMP3*, and *PCQAP/MED15* in Salivary DNA as a Quadruple Biomarker Panel for Early Detection of Oral and Oropharyngeal Cancers. Biomolecules. 2019 Apr 12;9(4):148. doi: 10.3390/biom9040148. PMID: 31013839; PMCID: PMC6523930. | Yes |  |
| 33 | Lepcha L, Sarma MP, Kataki AC, Wankhar W, Unni BG. Protein Profile of Human Saliva as a Predictive and Prognostic Tool for OSCC in Tamol Chewer's Population in Assam. Asian Pac J Cancer Prev. 2021 Jun 1;22(6):1837-1841. doi: 10.31557/APJCP.2021.22.6.1837. PMID: 34181340; PMCID: PMC8418859. | Yes |  |
| 34 | Ueda S, Goto M, Hashimoto K, Imazawa M, Takahashi M, Oh-Iwa I, Shimozato K, Nagao T, Nomoto S. Salivary *CPLANE1* Levels as a Biomarker of Oral Squamous Cell Carcinoma. Anticancer Res. 2021 Feb;41(2):765-772. doi: 10.21873/anticanres.14828. PMID: 33517281. | Yes |  |
| 35 | Triani M, Widodo HB, Novrial D, Agustina D, Nawangtantrini G. Salivary ki-67 and micronucleus assay as potential biomarker of OSCC in betel nut chewers. J Indian Acad Oral Med Radiol. 2021;33(2):146-51. | Yes |  |
| 36 | Smriti K, Ray M, Chatterjee T, Shenoy RP, Gadicherla S, Pentapati KC, Rustaqi N. Salivary MMP-9 as a Biomarker for the Diagnosis of Oral Potentially Malignant Disorders and Oral Squamous Cell Carcinoma. Asian Pac J Cancer Prev. 2020 Jan 1;21(1):233-238. doi: 10.31557/APJCP.2020.21.1.233. PMID: 31983189; PMCID: PMC7294014. | Yes |  |
| 37 | Patil MB, Lavanya T, Kumari CM, Shetty SR, Gufran K, Viswanath V, Swarnalatha C, Babu JS, Nayyar AS. Serum ceruloplasmin as cancer marker in oral pre-cancers and cancers. J Carcinog. 2021 Sep 30;20:15. doi: 10.4103/jcar.jcar_10_21. PMID: 34729047; PMCID: PMC8531576. | Yes |  |
| 38 | Ukey S, Jain A, Dwivedi S, Choudhury C, Vishnoi JR, Chugh A, Purohit P, Pareek P, Elhence P, Misra S, Sharma P. Study of MicroRNA (miR-221-3p, miR-133a-3p, and miR-9-5p) expressions in oral submucous fibrosis and squamous cell carcinoma. Indian J Clinic Biochem. 2023;38(1):73-82. | Yes |  |
| 39 | de Vicente JC, Rodríguez-Santamarta T, Rodrigo JP, Allonca E, Vallina A, Singhania A, Donate-Pérez Del Molino P, García-Pedrero JM. The Emerging Role of NANOG as an Early Cancer Risk Biomarker in Patients with Oral Potentially Malignant Disorders. J Clin Med. 2019 Sep 3;8(9):1376. doi: 10.3390/jcm8091376. PMID: 31484317; PMCID: PMC6780631. |  | Yes (Wrong study population) |
| 40 | Azeem MS, Yesupatham ST, Mohiyuddin SMA, Sumanth V, Ravishankar S. Usefulness of salivary sialic acid as a tumor marker in tobacco chewers with oral cancer. J Cancer Res Ther. 2020 Apr-Jun;16(3):605-611. doi: 10.4103/jcrt.JCRT_337_19. PMID: 32719275. | Yes |  |
| 41 | Juan YC, Su YF, Bai CH, Fan YC, Kuo TT, Ko HH, Peng HH, Chiang CP, Fwu CW, Cheng SJ. ZNF582 hypermethylation as a prognostic biomarker for malignant transformation of oral lesions. Oral Dis. 2023 Mar;29(2):505-514. doi: 10.1111/odi.13946. Epub 2021 Jul 1. PMID: 34145953. | Yes |  |

**Table S5. Quality appraisal outcomes of the appraised quantitative randomized studies using the Mixed Methods Appraisal Tool**

| **No.** | **Author (Year)** | **Study Design** | **Responses to the Appraisal Questions for Quantitative Randomized Studies** | | | | | | | **Scored Points (out of a Total of 7 Points)** | **Grade** |
| --- | --- | --- | --- | --- | --- | --- | --- | --- | --- | --- | --- |
|  |  |  | Are there clear research questions? | Do the collected data allow to address the research questions? | Is randomization appropriately performed? | Are the groups comparable at baseline? | Are there complete outcome data? | Are outcome assessors blinded to the intervention provided? | Did the participants adhere to the assigned intervention? |  |  |
|  | Nil. | Nil. | Nil. | Nil. | Nil. | Nil. | Nil. | Nil. | Nil. | Nil. | Nil. |
| **Yes – 1 point; No – 0 point; I can’t tell – 0.5 point; Above average – 4/7 points and above; Below average – 3/7 points and below** | | | | | | | | | | | |

**Table S6. Quality appraisal outcomes of the appraised quantitative non-randomized studies using the Mixed Methods Appraisal Tool**

| **No.** | **Author (Year)** | **Study Design** | **Responses to the Appraisal Questions for Quantitative Non-randomized Studies** | | | | | | | **Scored Points (out of a Total of 7 Points)** | **Grade** |
| --- | --- | --- | --- | --- | --- | --- | --- | --- | --- | --- | --- |
|  |  |  | Are there clear research questions? | Do the collected data allow to address the research questions? | Are the participants representative of the largest population? | Are measurements appropriate regarding both the outcome and intervention (or exposure)? | Are there complete outcome data? | Are the confounders accounted for in the design and analysis? | During the study period, is the intervention administered (or exposure occurred) as intended? |  |  |
|  | Patel et al. (2021) | Cohort study (Prospective) | Yes | Yes | Yes | Yes | Yes | Yes | Yes | 7/7 | Above Average |
|  | Sajid et al. (2023) | Cohort study (Prospective) | Yes | Yes | I can’t tell | Yes | I can’t tell | No | Yes | 5/7 | Above Average |
|  | Chen et al. (2021) | Case-control study | Yes | Yes | I can’t tell | Yes | Yes | Yes | Yes | 6.5/7 | Above Average |
|  | Singh et al., (2018) | Case-control study | Yes | Yes | I can’t tell | Yes | Yes | No | Yes | 5.5/7 | Above Average |
|  | Ren et al., (2014) | Case-control study | Yes | Yes | I can’t tell | Yes | Yes | No | Yes | 5.5/7 | Above Average |
|  | Rochefort et al., (2022) | Cohort study (Prospective) | Yes | Yes | I can’t tell | Yes | Yes | No | Yes | 5.5/7 | Above average |
|  | Amer et al. (2018) | Cohort study (Prospective) | Yes | Yes | I can’t tell | Yes | Yes | Yes | Yes | 6.5/7 | Above Average |
|  | Huang et al. (2014) | Case–control study | Yes | Yes | I can’t tell | Yes | Yes | No | Yes | 5.5/7 | Above Average |
|  | Aghiorghiesei et al., (2022) | Case-control study | Yes | Yes | I can’t tell | Yes | Yes | No | I can’t tell | 5/7 | Above Average |
|  | Bhuvaneswari et al. (2021) | Cohort study (Prospective) | Yes | Yes | I can’t tell | Yes | Yes | Yes | Yes | 6.5/7 | Above Average |
|  | Rezazadeh et al. (2017) | Cohort study (Prospective) | Yes | Yes | I can’t tell | Yes | Yes | Yes | Yes | 6.5/7 | Above Average |
|  | Vishwakarma et al., (2020) | Case control study | Yes | Yes | I can’t tell | Yes | Yes | Yes | Yes | 6.5/7 | Above average |
|  | Arunkumar et al., (2017) | Cohort study (Prospective) | Yes | Yes | I can’t tell | Yes | Yes | Yes | Yes | 6.5/7 | Above Average |
|  | Su et al. (2018) | Case-control study | Yes | Yes | Yes | Yes | Yes | Yes | Yes | 7/7 | Above Average |
|  | Cheng et al. (2016) | Cross-sectional analytical study | Yes | Yes | I can’t tell | Yes | Yes | Yes | Yes | 6.5/7 | Above Average |
|  | Tandon et al., (2017) | Case-control study | Yes | Yes | Yes | Yes | Yes | No | Yes | 6/7 | Above average |
|  | Mondal et al. (2013) | Cohort study (Prospective) | Yes | Yes | I can’t tell | Yes | I can’t tell | Yes | Yes | 6/7 | Above Average |
|  | Sun et al., (2023) | Cohort study (Retrospective) | Yes | Yes | I can’t tell | Yes | Yes | Yes | Yes | 6.5/7 | Above Average |
|  | D’Cruz et al. (2021) | Cohort study (Prospective) | Yes | Yes | I can’t tell | Yes | I can’t tell | No | Yes | 5/7 | Above Average |
|  | Sawant et al., (2023) | Cohort study (Prospective) | Yes | Yes | I can’t tell | Yes | Yes | Yes | Yes | 6.5/7 | Above Average |
|  | Goel et al., (2021) | Case-control study | Yes | Yes | I can’t tell | Yes | Yes | No | Yes | 5.5/7 | Above Average |
|  | Lepcha et al., (2021) | Cohort study (Prospective) | Yes | Yes | No | Yes | I can’t tell | No | I can’t tell | 4/7 | Above Average |
|  | Liyanage et al. (2019) | Cross-sectional analytical study | Yes | Yes | I can’t tell | Yes | Yes | Yes | Yes | 6/7 | Above Average |
|  | Ueda et al., (2021) | Case-control study | Yes | Yes | I can’t tell | Yes | Yes | Yes | Yes | 6.5/7 | Above Average |
|  | Triani et al., (2021) | Cohort study (Prospective) | Yes | Yes | I can’t tell | Yes | Yes | Yes | Yes | 6.5/7 | Above Average |
|  | Smriti et al., (2020) | Cohort study (Prospective) | Yes | Yes | I can’t tell | Yes | Yes | No | Yes | 5.5/7 | Above Average |
|  | Patil et al. (2021) | Case-control study | Yes | Yes | I can’t tell | Yes | Yes | Yes | Yes | 6.5/7 | Above Average |
|  | Ukey et al., (2023) | Case control study | Yes | Yes | I can’t tell | Yes | Yes | No | Yes | 5.5/7 | Above average |
|  | Azeem et al. (2020) | Cohort study (Prospective)? | Yes | Yes | I can’t tell | Yes | Yes | No | Yes | 5.5/7 | Above Average |
|  | Juan et al., (2023) | Case–control study? | Yes | Yes | Yes | Yes | Yes | Yes | Yes | 7/7 | Above Average |
|  | Chaudhari et al. (2016) | Case-control study | Yes | Yes | I can’t tell | Yes | Yes | Yes | Yes | 6.5/7 | Above Average |
|  | Bose et al. (2013) | Cohort study (Prospective) | Yes | Yes | I can’t tell | Yes | I can’t tell | No | Yes | 5/7 | Above Average |
|  | Goyal (2020) | Cohort study (Prospective) | Yes | Yes | Yes | Yes | Yes | Yes | Yes | 7/7 | Above Average |
|  | Oh et al., (2020) | Case-control study | Yes | Yes | I can’t tell | Yes | Yes | No | I can’t tell | 5/7 | Above Average |
|  | Dineshkumar et al. (2016) | Case-control study | Yes | Yes | I can’t tell | Yes | Yes | Yes | Yes | 6.5/7 | Above Average |
|  | Deepthi et al. (2019) | Case-control study | Yes | Yes | I can’t tell | Yes | Yes | No | Yes | 5.5/7 | Above Average |
| **Yes – 1 point; No – 0 point; I can’t tell – 0.5 point; Above average – 4/7 points and above; Below average – 3/7 points and below** | | | | | | | | | | | |

**Table S7. Quality appraisal outcomes of the appraised quantitative descriptive study using the Mixed Methods Appraisal Tool**

| **No.** | **Author (Year)** | **Study Design** | **Responses to the Appraisal Questions for Quantitative Descriptive Studies** | | | | | | | **Scored Points (out of a Total of 7 Points)** | **Grade** |
| --- | --- | --- | --- | --- | --- | --- | --- | --- | --- | --- | --- |
|  |  |  | Are there clear research questions? | Do the collected data allow to address the research questions? | Is the sampling strategy relevant to address the research question? | Is the sample representative of the target population? | Are the measurements appropriate? | Is the risk of nonresponse bias low? | Is the statistical analysis appropriate to answer the research question? |  |  |
|  | Nil. | Nil. | Nil. | Nil. | Nil. | Nil. | Nil. | Nil. | Nil. | Nil. | Nil. |
| **Yes – 1 point; No – 0 point; I can’t tell – 0.5 point; Above average – 4/7 points and above; Below average – 3/7 points and below** | | | | | | | | | | | |

**Table S8. Quality appraisal outcomes of the appraised qualitative study using the Mixed Methods Appraisal Tool**

| **No.** | **Author (Year)** | **Study Design** | **Responses to the Appraisal Questions for Qualitative Studies** | | | | | | | **Scored Points (out of a Total of 7 Points)** | **Grade** |
| --- | --- | --- | --- | --- | --- | --- | --- | --- | --- | --- | --- |
|  |  |  | Are there clear research questions? | Do the collected data allow to address the research questions? | Is the qualitative approach appropriate to answer the research question? | Are the qualitative data collection methods adequate to address the research question? | Are the findings adequately derived from the data? | Is the interpretation of results sufficiently substantiated by data? | Is there coherence between qualitative data sources, collection, analysis and interpretation? |  |  |
|  | Nil. | Nil. | Nil. | Nil. | Nil. | Nil. | Nil. | Nil. | Nil. | Nil. | Nil. |
| **Yes – 1 point; No – 0 point; I can’t tell – 0.5 point;** **Above average – 4/7 points and above; Below average – 3/7 points and below** | | | | | | | | | | | |
